# The EHA Research Roadmap: Malignant Lymphoid Diseases

**DOI:** 10.1097/HS9.0000000000000726

**Published:** 2022-05-19

**Authors:** Martin Dreyling, Marc André, Nicola Gökbuget, Hervé Tilly, Mats Jerkeman, John Gribben, Andrés Ferreri, Pierre Morel, Stephan Stilgenbauer, Christopher Fox, José Maria Ribera, Sonja Zweegman, Igor Aurer, Csaba Bödör, Birgit Burkhardt, Christian Buske, Maria Dollores Caballero, Elias Campo, Bjoern Chapuy, Andrew Davies, Laurence de Leval, Jeanette Doorduijn, Massimo Federico, Philippe Gaulard, Francesca Gay, Paolo Ghia, Kirsten Grønbæk, Hartmut Goldschmidt, Marie-Jose Kersten, Barbara Kiesewetter, Judith Landman-Parker, Steven Le Gouill, Georg Lenz, Sirpa Leppä, Armando Lopez-Guillermo, Elizabeth Macintyre, Maria Victoria Mateos Mantega, Philippe Moreau, Carol Moreno, Bertrand Nadel, Jessica Okosun, Roger Owen, Sarka Pospisilova, Christiane Pott, Tadeusz Robak, Michelle Spina, Kostas Stamatopoulos, Jan Stary, Karin Tarte, Allessandra Tedeschi, Catherine Thieblemont, Ralf Ulrich Trappe, Lorenz H. Trümper, Gilles Salles

**Affiliations:** 1 Department of Medicine III, LMU Hospital, Munich, Germany; 2 Université Catholique de Louvain, CHU UcL Namur, Yvoir, Belgium; 3 Department of Medicine II, Hematology/Oncology, University Hospital Frankfurt, Goethe University, Frankfurt, Germany; 4 INSERM U1245, Department of Hematology, Centre Henri Becquerel and Université de Rouen, France; 5 Skåne University and Lund University, Lund, Sweden; 6 Barts Cancer Institute, Queen Mary University of London, United Kingdom; 7 Lymphoma Unit, Department of Onco-hematology, IRCCS San Raffaele Scientific Institute, Milano, Italy; 8 Service Hematologie Clinique Therapie Cellulaire, CHU Amiens Picardie, Amiens, France; 9 Comprehensive Cancer Center Ulm (CCCU), Sektion CLL Klinik für Innere Medizin III, Universität Ulm, Germany; 10 School of Medicine, University of Nottingham, United Kingdom; 11 Clinical Hematology Department, ICO-Hospital Germans Trias i Pujol, Josep Carreras Research Institute, Badalona, Spain; 12 Amsterdam UMC, Vrije Universiteit Amsterdam, Cancer Center Amsterdam, the Netherlands; 13 University Hospital Centre Zagreb and Medical School, University of Zagreb, Croatia; 14 HCEMM-SE Molecular Oncohematology Research Group, Department of Pathology and Experimental Cancer Research, Semmelweis University, Budapest, Hungary; 15 Experimentelle und Translationale päd. Hämatologie u Onkologie, Leitung der Bereiche Lymphome und Stammzelltransplantation, Universitätsklinikum Münster (UKM), Klinik für Kinder- und Jugendmedizin, Pädiatrische Hämatologie und Onkologie, Munich, Germany; 16 Institute of Experimental Cancer Research, CCC Ulm, University Hospital Ulm, Germany; 17 Clinical and Transplant Unit, University Hospital of Salamanca, Spain; 18 Department of Medicine at the University of Salamanca, Spain; 19 El Instituto de Investigación Biomédica de Salamanca (IBSAL), Salamanca, Spain; 20 Institut d’Investigacions Biomèdiques August Pi i Sunyer (IDIBAPS), Barcelona, Spain; 21 Department of Hematology, Oncology and Tumor Immunology, Charité, University Medical Center Berlin, Campus Benjamin Franklin, Berlin, Germany; 22 Berlin Institute of Health, Berlin, Germany; 23 Southampton NCRI/UK Experimental Cancer Medicines Centre, Faculty of Medicine, University of Southampton, United Kingdom; 24 Department of Laboratory Medicine and Pathology, Institute of Pathology, Lausanne University Hospital and Lausanne University, Lausanne, Switzerland; 25 Department of Hematology, Erasmus MC Cancer Institute, University Medical Center Rotterdam, Rotterdam, the Netherlands; 26 Città di Lecce Hospital, GVM Care & Research, Lecce, Italy; 27 Département de Pathologie, Hôpital Henri Mondor, AP-HP, Créteil, France; 28 Clinical Trial Unit, Division of Hematology 1, AOU Città Della Salute e Della Scienza, University of Torino, Italy; 29 Università Vita Salute San Raffaele and IRCCS Ospedale San Raffaele, Milano, Italy; 30 Department of Hematology, Rigshospitalet, Copenhagen, Denmark; 31 Biotech Research & Innovation Centre (BRIC), University of Copenhagen, Denmark; 32 University Hospital Heidelberg, Internal Medicine V and National Center for Tumor Diseases (NCT), Heidelberg, Germany; 33 Department of Hematology, Amsterdam UMC, University of Amsterdam, Cancer Center Amsterdam and LYMMCARE, Amsterdam, the Netherlands; 34 Department of Medicine I, Division of Oncology, Medical University of Vienna, Austria; 35 Pediatric Hematology Oncology, Sorbonne Université APHP/hôpital A Trousseau, Paris, France; 36 Service d’Hématologie, Clinique du Centre Hospitalier Universitaire (CHU) de Nantes, France; 37 Medical Department A for Hematology, Oncology and Pneumology, University Hospital Münster, Germany; 38 University of Helsinki and Helsinki University Hospital Comprehensive Cancer Centre, Helsinki, Finland; 39 Department of Hematology, Hospital Clínic de Barcelona, Spain; 40 Onco-hematology, Université de Paris and Necker-Enfants Malades Hospital, Assistance Publique-Hôpitaux de Paris, France; 41 Complejo Asistencial Universitario de Salamanca, IBSAL, CIC, Universidad de Salamanca, Ciberonc, Salamanca, Spain; 42 Hematology Department, University Hospital Hotel-Dieu, Nantes, France; 43 Hospital de la Santa Creu I Sant Pau, Autonomous University of Barcelona, Spain; 44 Aix Marseille Univ, CNRS, INSERM, CIML, Marseille, France; 45 Centre for Haemato-Oncology, Barts Cancer Institute, Queen Mary University of London, United Kingdom; 46 St James’s Institute of Oncology, Leeds, United Kingdom; 47 Department of Internal Medicine—Hematology and Oncology and Department of Medical Genetics and Genomics, Faculty of Medicine, Masaryk University and University Hospital Brno, Czech Republic; 48 Klinisch-experimentelle Hämatologie, Medizinische Klinik II, Hämatologie und Internistische Onkologie, Universitätsklinikum Schleswig-Holstein, Campus Kiel, Germany; 49 Medical University of Lodz, Poland; 50 Division of Medical Oncology and Immune-related Tumors, National Cancer Institute, Aviano, Italy; 51 Institute of Applied Biosciences, Centre for Research and Technology Hellas, Thessaloniki, Greece; 52 Department of Pediatric Hematology and Oncology 2nd Faculty of Medicine, Charles University Prague University Hospital, Prague, Czech Republic; 53 Immunology and Cell Therapy Lab at Rennes University Hospital, Rennes, France; 54 Department of Hematology, Niguarda Hospital Milano, Italy; 55 Department of Hemato-Oncology, Saint-Louis Hospital, Assistance Publique-Hôpitaux de Paris (AP-HP), Paris, France; 56 Department of Internal Medicine II: Haematology and Oncology, DIAKO Hospital Bremen, Germany; 57 Hematology and Medical Oncology, University Medicine Goettingen, Germany; 58 Lymphoma Service, Department of Medicine, Memorial Sloan Kettering Cancer Center, Weill Cornell Medicine, New York, NY, USA

*In 2016, the European Hematology Association (EHA) published the EHA Roadmap for European Hematology Research*^1^ *aiming to highlight achievements in the diagnostics and treatment of blood disorders and to better inform European policy makers and other stakeholders about the urgent clinical and scientific needs and priorities in the field of hematology. Each section was coordinated by 1 to 2 section editors who were leading international experts in the field. In the 5 years that have followed, advances in the field of hematology have been plentiful. As such, EHA is pleased to present an updated Research Roadmap, now including 11 sections, each of which will be published separately. The updated EHA Research Roadmap identifies the most urgent priorities in hematology research and clinical science, therefore supporting a more informed, focused, and ideally a more funded future for European hematology research. The 11 EHA Research Roadmap sections include Normal Hematopoiesis; Malignant Lymphoid Diseases; Malignant Myeloid Diseases; Anemias and Related Diseases; Platelet Disorders; Blood Coagulation and Hemostatic Disorders; Transfusion Medicine; Infections in Hematology; Hematopoietic Stem Cell Transplantation; CAR-T and Other Cell-based Immune Therapies; and Gene Therapy.*

Malignant lymphoid diseases represent the most frequent hematologic malignancies, with an age-adjusted estimated incidence of 24.5 per 100,000 inhabitants in Europe,^2^ and are associated with significant mortality^3^ and morbidity. This disease group is highly heterogeneous in terms of frequency, epidemiology, biology, genetic abnormalities, and outcome. Although in a way all individual lymphoma subtypes may be characterized as rare diseases, some of them are relatively common, for example, multiple myeloma, chronic lymphocytic leukemia (CLL), diffuse large B-cell lymphomas (DLBCLs), follicular lymphomas (FLs), and Hodgkin lymphomas (HLs). Others are less common, for example, mantle cell lymphoma (MCL), acute lymphoblastic leukemia (ALL), T-cell lymphoma, and mucosa-associated lymphoid tissue (MALT) lymphoma, Waldenstrom’s Macroglobulinemia, or even very rare, for example, some subsets of marginal zone lymphomas (MZLs) and HIV-associated lymphoma. After the progress made in the morphological classification of these tumors in the 1990s, the advent of large-scale genomic approaches enabled identification of multiple molecular subsets, which may further subdivide the different entities in multiple rare diseases.^4–6^ These achievements justify the need for European-based epidemiological studies and contributions to the InterLymph consortium^7^ to investigate the role of environmental and lifestyle factors, which, in the context of inherited genetic background, may favor the development of these malignancies.

Significant progress was also made in unraveling key biological features of these diseases, including (1) the more precise delineation of intrinsic genetic defects in tumor cells, delineation still ongoing with next-generation sequencing (NGS) approaches^6,8,9^; (2) the growing understanding of the complex interplays between malignant cells and their microenvironment, which is especially critical in these diseases arising in lymphoid organs^10^; and (3) the emerging identification of constitutional genetic traits associated with an increased susceptibility to develop these malignancies.^11,12^ Although several European groups have already made outstanding contributions to this field, in part within large international consortia, further achievements will only be possible if major investments can be realized. These should particularly focus on establishing new cellular and animal models which are critically rare in the field of mature lymphoid malignancies to better understand how these diseases develop and for preclinical assessment of new therapeutic agents.

Despite important advances in the past few years,^13^ the survival of patients with lymphoid malignancies remains unsatisfactory. This is true for the most aggressive malignancies (eg, ALLs, T-cell lymphomas, and some forms of DLBCL), which still are frequently fatal. In addition, the lack of cure in patients with multiple myeloma or indolent lymphoma is equally challenging. Furthermore, short- or long-term morbidities such as infertility, secondary malignancies, as well as cardiac, pulmonary, renal, or neurological dysfunction are associated with intensive treatment in HL or DLBCL. Chronic exposure to therapeutic agents such as in indolent lymphoma and CLL also represents a health burden for patients, as well as an increasingly relevant economic burden for the European Union.^14,15^ Attention to malignancies occurring in elderly patients should also be considered in this regard given the fact that life expectancies continue to grow.

European co-operative groups have been leading clinical research in lymphoid malignancies in the past decades. Progress is being made in investigating the role of targeted agents in well-characterized molecular subsets. The number of new therapeutic agents under development in this field demands further academic research collaboration. For example, analyzing the medico-economic impacts of patient management should clarify the costs and benefits of novel therapeutic strategies, including those related to public health economics. These groups also need further support in their translational research activities, especially in their efforts to constitute and analyze large biobanks with high-quality clinical annotations. Efforts should also aim to eliminate the different outcomes observed in different parts of Europe and to improve patients’ survival and quality of life.

## HODGKIN LYMPHOMA

### Introduction

Classical HL is a highly curable disease and for both localized and advanced-stage diseases, >90% of patients are alive 5 years after diagnosis. During their follow-up, however, a significant proportion of these young patients experience serious long-term toxicities related to the treatments of lymphoma. The reduction of long-term, treatment-related toxicities remains the goal of actual clinical trials.

### European research contributions

Based on the seminal work of Gallamini et al,^16^ it appears that an early PET scan (e-PET) performed after 2–3 cycles of ABVD was able to segregate patients into two categories; early PET negative patients with an excellent prognosis and a possibility to reduce the amount of treatment; and early PET-positive patients with a worse prognosis suggesting no reduction but possibly intensification of treatment.

In early stage HL, three European studies based on this approach were performed and published in the last 5 years.^17–19^ In early stage favorable HL, the objective was to avoid radiotherapy in e-PET negative patients. All three trials were not able to demonstrate the noninferiority of the no radiotherapy arm in terms of progression-free survival (PFS), but the overall survival (OS) was excellent and did not show any difference. Omission of radiotherapy is at the price of some reduction in tumor control and should be balanced with the expected individual risk associated with radiotherapy.

In early stage intermediate/unfavorable disease, the same strategy was applied.^18,20^ In the HD17 study, noninferiority of the no radiotherapy arm was demonstrated and in the H10 study a 2.5% of difference of PFS was observed between the 2 arms suggesting that radiotherapy can be omitted in this situation. For e-PET-positive patients, only the H10 study evaluated early intensification of chemotherapy and demonstrated a better PFS with this strategy; improvement of OS was of borderline significance.

In advanced-stage HL, three randomized studies evaluated reduction of the amount of treatment based on an e-PET evaluation.^21–23^ All these three studies were successful and demonstrated that a reduction of the amount of treatment given is possible when an e-PET negativity is reached after 2 cycles of chemotherapy. For e-PET negative patients, omission of Bleomycin, reduction to 4 cycles of escBEACOPP, or de-escalation to ABVD were validated by these three trials.

In adolescents and young adults (AYA), to limit gonadal damage and second malignancies, the European trial EuroNet PHL C1^24^ lead to the replacement of procarbazine with dacarbazine and to restriction of RT indications to patients with an adequate response after 2 first cycles of OEPA. EuroNet PHL C2 explores moderate treatment intensification in order to further limit RT indications.

Finally, the incorporation of Brentuximab Vedotin (BV) and checkpoint inhibitors in the ABVD regimen was evaluated recently. Two European studies^25,26^ showed that replacing Bleomycin by one of these two drugs could be achieved safely. BV-AVD increased e-PET negativity compared with ABVD.

### Proposed research for the Roadmap

Individualization of treatment should be pursued in the context of PET-adapted therapy. Besides early PET negativity, total metabolic tumor volume appears to be an important prognostic factor and should be tested for better tailoring the risk-adapted treatment strategy.

Another possible important avenue for future research is the detection of tumor DNA in the blood of the patients. Its potential use to identify some baseline prognostic factors, monitor treatment, and detect early relapses warrant future development. In this context, systematic banking of tumor and plasma samples, such as PET images is recommended in future European trials. BV and checkpoints inhibitors have a definitive place in refractory/relapsed HL lymphoma, their possible integration into first line for selected patients need still to be demonstrated in future phase III trials.

### Anticipated impact of the research

Reducing toxicities and possibly improving the high-cure rate will remain the goal of our future strategies and probably led to even more individualized therapy, incorporating PET, cell-free DNA, and possibly new drugs. As the costs of these new tools are elevated, they will probably not apply to each patient and be restricted to a subset of HL. Special attention should be given to the dissemination of these expensive innovations (and future ones such as CART-T cells) in all countries in Europe.

## ACUTE LYMPHOBLASTIC LEUKEMIA

### Introduction

Acute lymphoblastic leukemia is a life-threatening disease affecting children and adults. Treatment consists of combination chemotherapy, with allogeneic hematopoietic stem cell transplantation (HSCT) restricted to high-risk or relapsed ALL. Five-year event-free survival is correlated to age and in contemporary treatment, protocols reach over 80% for children and 40%–70% for adults, due to the increased incidence of poor prognostic features and lower tolerability of intensive chemotherapy in older patients.^1^

### European research contributions

The treatment of ALL in Europe is undertaken by national study groups and international consortia such as BFM/AEIOP and ALL-Together for children and EWALL for adults. National study group clinical databases, reference laboratories, or associated biobanks are essential for research on disease biology and prognostication and are sources of reliable real-world data. European standardization for monitoring of minimal residual disease (MRD) by the EuroMRD/ESLHO group made risk-based individual treatment modification possible. As a basis for intergroup trials, consensus definitions for adverse events and treatment response have been developed for pediatric ALL.^27,28^ Gene expression profiling and sequencing have identified many new subtypes of B-cell precursor (BCP) and T-cell lineage ALL; their number has complicated both European standardization and risk-based personalized therapy.

Immune therapies with bispecific antibodies and conjugated antibodies for BCP-ALL have replaced standard of care chemotherapy in R/R ALL,^29^ led for the first time to marketing authorization for MRD-positive ALL^30^ and are increasingly being tested in first-line trials. CAR-T cell products became available within clinical trials and with the marketed product for patients younger than 25 years. Further trials are directed to the clinical evaluation of inhibitors directed to BCR-ABL-like or JAK-class fusion-gene subgroups.^31^ On the other hand, the clinical impact of intensive conventional treatments like HSCT with TBI-based conditioning has been underlined.^32^

### Proposed research for the Roadmap

Evaluation of the prognostic impact of the myriad oncogenic subgroups in modern, MRD-risk stratified, treatment protocols is challenging. It is essential, in addition to identifying and unraveling potential prognostic lesions, to increasingly focus on functional studies addressing tumor dependency of new lesions (including those found in subclones) in relevant models. In vitro testing strategies to select compounds for individual refractory patients are emerging^33^ and require European concertation and adaptation of data management, clinical trial design, and drug accessibility circuits. Epigenetic landscapes are more accessible for the identification of actionable targets, such as hypomethylating agents^34^ and multiparameter prognostic scores will increasingly combine clinical and molecular features.^35^ Our knowledge about the supportive (and protective) role of the bone marrow microenvironment should also be expanded.^1^ It is also essential to address future ALL classification, which needs to integrate molecular oncogenic characteristics and next-generation MRD and requires standardization of the optimal diagnostic standards.

Late effects of treatment, such as osteonecrosis,^36^ are increasingly relevant with improved ALL survival, requiring joint efforts of pediatric and adult study groups to better understand biology, improve surveillance and define potential treatment modifications. Age is one of the most important prognostic factors for ALL. Uncertainty persists on which types of pediatric-based therapies are tolerated in different age groups so, at least, clear reporting standards are necessary. The development of innovative treatment strategies for older patients can help younger patients and vice versa.

Increasing regulatory issues are emerging regarding drug development, marketing authorization, and reimbursement for new drugs in adult ALL, which are treated with complex combination therapies and management strategies. Support structures developed for pediatric ALL^1^ should be extended to adult ALL, in collaboration between national competent authorities, Institutional Review Boards, pharmaceutical companies, and academic study groups. The evaluation of new compounds in rare molecular subgroups of ALL will require European-level collaboration with the European Medical Agency and intergroup data-sharing, as in the Harmony IMI initiative, https://www.harmony-alliance.eu. New strategies for clinical trial design must be developed in concertation with patient representatives, and there is an urgent need to support the successful infrastructure of European academic multicenter study groups through funding programs.

In the next years, these challenges will become evident for the integration of new monoclonal antibodies and cell therapies into first line. Several clinical trials are ongoing in Europe and worldwide requiring harmonization to enable future meta-analyses.^37–39^ The scientific questions do not only focus on the impact of a single compound but new combination strategies and the role of current drugs, risk stratifications, and approaches like HSCT. One major research question will certainly focus on the future role of HSCT in adult and pediatric ALL.

For Ph+ ALL the selection of TKIs, the efficacy of chemotherapy-free regimens,^40^ indications for change of TKI, the impact of MRD measured with different methods, the role of immunotherapies, and HSCT will be important research questions.

One priority for the research agenda is the management of T-ALL and T-LL. Less progress has been made regarding the biologic characterization and prognostic classification, with a relative paucity of promising new compounds^41^ and immunotherapeutic targets. Whereas the overall prognosis of T-ALL is quite favorable, new approaches are urgently required for poor prognostic subgroups since survival after relapse is rare.

### Anticipated impact of the research

Future treatment will still be based on current very successful standards, however, targeted therapies including immunotherapies will be increasingly implemented. These should improve the prognosis of high-risk patients but also significantly reduce treatment-related morbidity for patients of all ages, and especially for long-term survivors of childhood ALL. In addition, individualized drug dosing may prevent underdosing and hence may reduce the risk of relapse, while preventing over-dosing and associated toxic side effects.

## DIFFUSE LARGE B-CELL LYMPHOMA AND BURKITT LYMPHOMA

### Introduction

**Figure 1.**
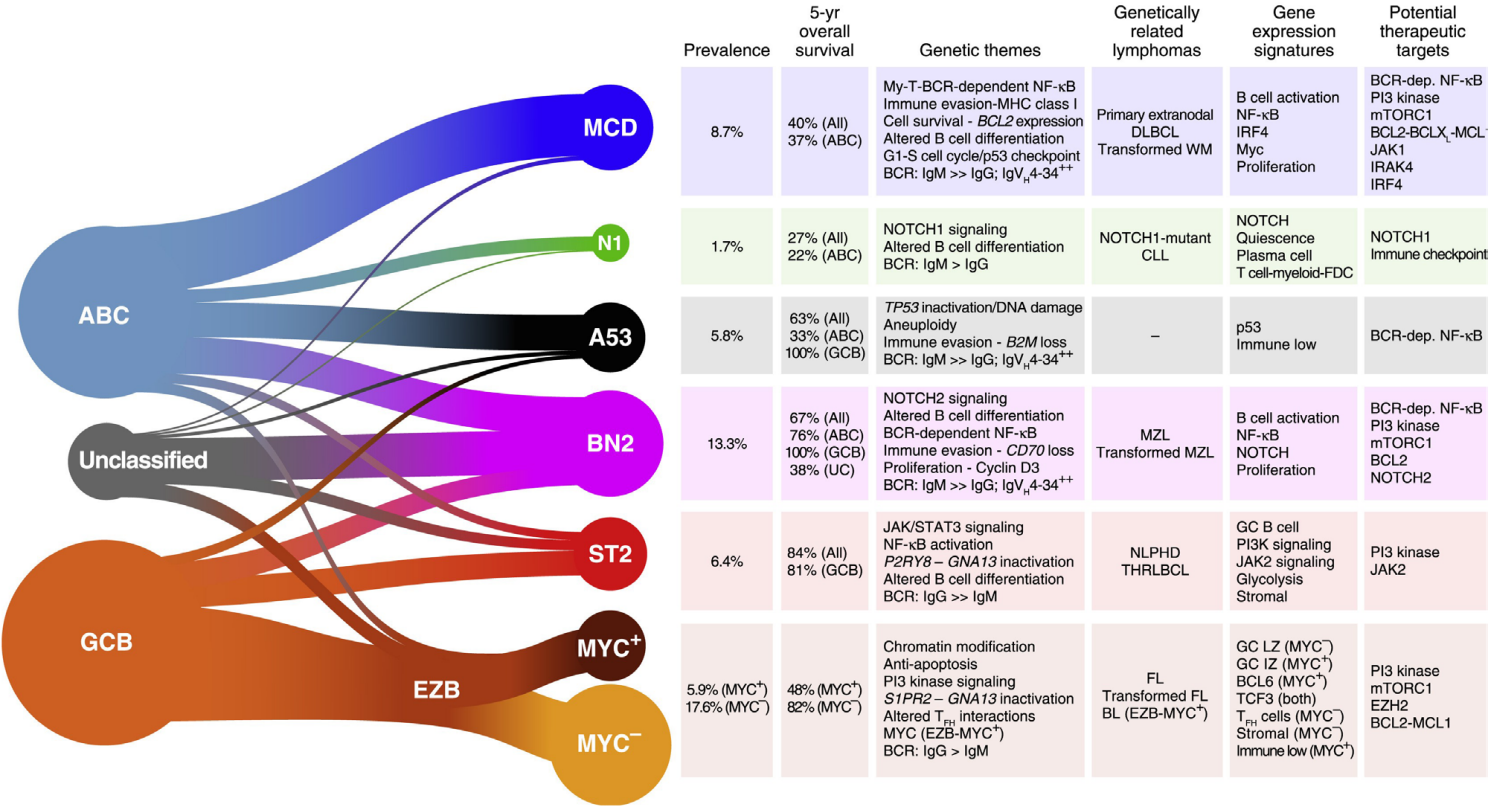
**Implications of the DLBCL genetic subtypes for pathogenesis and therapy.** Summary of the relationship between DLBCL COO subgroups and the genetic subtypes (left). The genetic themes, phenotypic attributes, clinical correlates, and treatment implications of each subtype are shown at right. Prevalences were estimated using the NCI cohort, adjusting for a population-based distribution of COO subgroups (see STAR Methods).^42^ dep. = dependent; DLBCLs = diffuse large B-cell lymphomas; FDC = follicular dendritic cell; IZ = intermediate zone; LZ = light zone. Reprinted from: *Cancer Cell*, Vol 37/issue 4, Authors Wright GW, et al, A Probabilistic Classification Tool for Genetic Subtypes of Diffuse Large B Cell Lymphoma with Therapeutic Implications, Pages 551-598, Copyright 2020, with permission from Elsevier.

Diffuse large B-cell lymphoma (DLBCL) is the most common clinically aggressive lymphoid neoplasm. In addition to the most common DLBCL “not otherwise specified” type, comprising germinal center B-cell, activated B-cell like subtypes, the 2016 WHO classification recognizes specific variants (eg, T-cell/histiocyte-rich large B-cell lymphoma, EBV-positive DLBCL) and high-grade B-cell lymphoma with *MYC* and *BCL2* or *BCL6* translocations as a separate entity (Figure 1).^42^

### European research contributions

European researchers have contributed to international efforts redefining molecular classifications of aggressive B-cell lymphoma beyond the transcriptionally cell of origin classification to identify genetically defined subtypes that suggest a specific lymphomagenesis, corresponding to a distinct outcome and allowing to consider different treatment approaches.^43,44^ Additionally, European hematologists participated in major clinical trials aiming at improving first-line treatment of DLBCL.^45–47^Until now, however, intensification of treatment, the addition of maintenance therapy, the introduction of immunomodulatory or novel-targeted agents have neither improved outcomes in unselected patient populations nor identified selected subgroups of patients, who could benefit from a new combination. On the other side, these studies have been able to reduce toxicity without affecting efficacy in patients at low-risk^48^ and new initiatives have been proposed to guide treatment strategy based on early response, particularly based on PET-CT.^49,50^ In addition, several European studies evaluated new agents or new combinations.^51–55^ The development of CAR-T cell therapy in relapsed and refractory DLBCL was a revolution initiated in the United States, but the networking of European treatment centers has made it possible to observe a large number of patients and continue to evaluate this method in real life.

In AYA, DLBCL is the second most common aggressive B-NHL. Outcome for DLBCL patients improved in recent clinical trials using Bukitt-type treatment regimen.^56^ Analogue to adults, genetic sub-classification will support subgroup identification and treatment modulation.^57^

### Proposed research for the Roadmap

The Roadmap is in line with the one proposed five years ago. We have now tools that will allow to further investigate mechanisms of DLBCL pathogenesis including novel models and leverage high-throughput functional screens (genetic and pharmacological) to identify unappreciated vulnerabilities and guide future drug development. Recent advances in understanding of the genetic heterogeneity of DLBCL that led to the definition of molecular subgroups could be accessible to existing or newly designed targeted therapies. The ability to recognize these subgroups in a reliable, reproducible but also timely manner will be an important challenge to introduce these drugs in the first-line treatment in AYA. The availability of circulating tumor DNA (ctDNA) could help to characterize the genomic profile of the disease.

The next wave of technologies will allow to capture single cell and spatial heterogeneity and to comprehensively understand the role of the tumor microenvironment and the “fitness” of the immune system. The knowledge of the immunological synapse will be invaluable to better address treatment with immunomodulatory drugs, bispecific antibodies, and CAR-T cells. These data should allow the construction of a new prognostic indices integrating multiomics and clinical characteristics.

It will be of interest to monitor early treatment response to guide subsequent treatment, either in the direction of a reduction or toward a different strategy. The combined use of PET-CT and ctDNA will be key tools for this longitudinal assessment.

The disappointing results of large studies aiming to improve first-line treatment and the detected genetic and clinical heterogeneity of DLBCL underline the limit of “one size fits all” in this disease. Tailoring therapeutic approaches of specific clinical, morphological, and molecular entities will require collaboration of European national co-operative groups and evolution of the methodology how clinical trials are performed. Similarly, real-life studies, based on the observation of a large number of patients, will be very useful in the evaluation of new treatments, especially those that are very expensive.

### Anticipated impact of the research

These research directions aim at a better understanding of biology and a better management of patients with DLBCL. The personalized treatment, more effective and safer, for DLBCL patients is not yet in our hands. A close collaboration between investigators, academic researchers, pharmaceutical companies, and patient associations will be necessary to achieve this goal and allow this progress to be shared throughout Europe.

## MANTLE CELL LYMPHOMA

### Introduction

**Figure 2.**
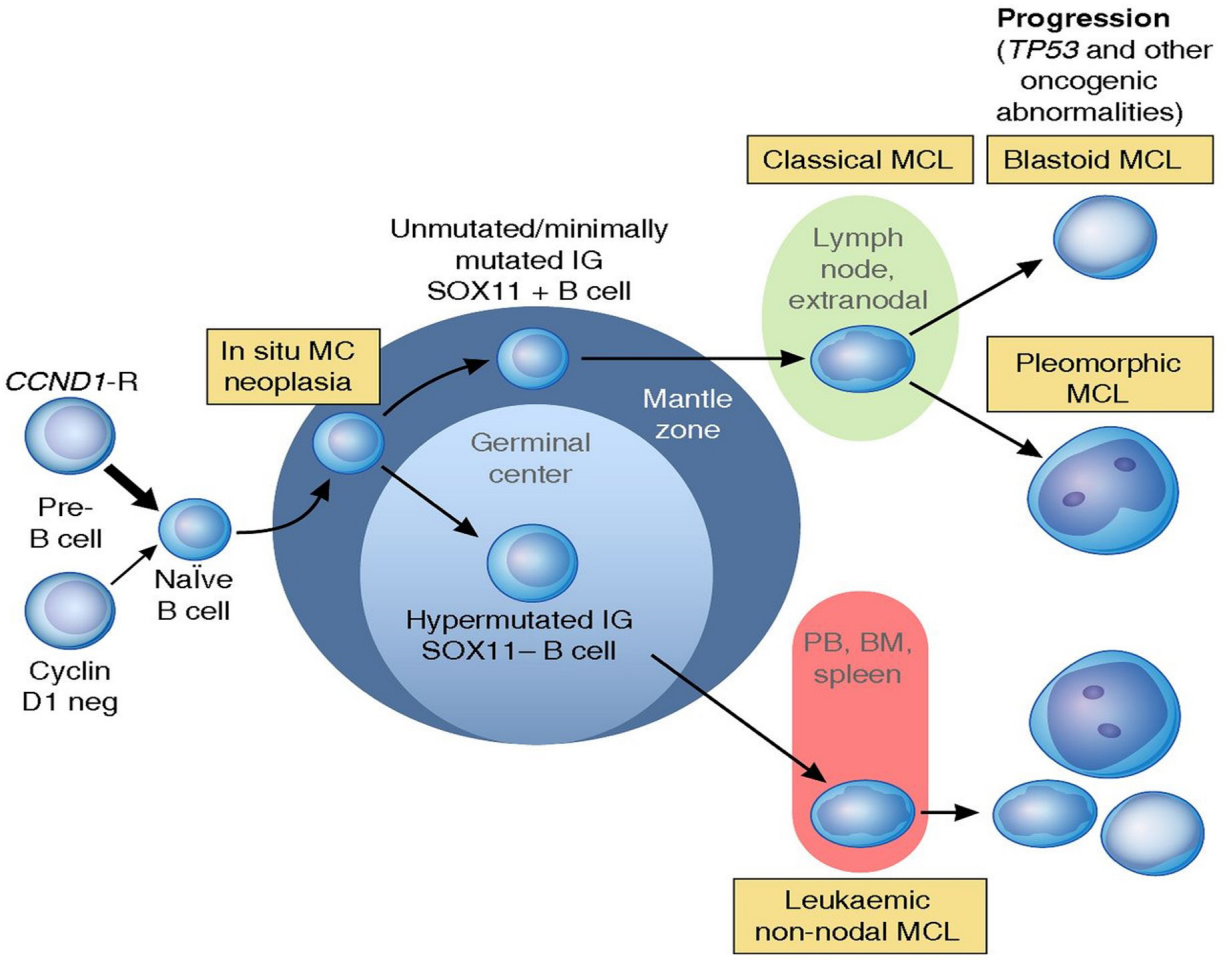
**Proposed model of molecular pathogenesis in the development and progression of major subtypes of mantle cell lymphoma.** ^58^ Reprinted from *Blood*, Vol 127/issue 20, Authors Swerdlow SH, et al, The 2016 revision of the World Health Organization classification of lymphoid neoplasms, Pages 2376-2390, Copyright 2016, with permission from Elsevier and/or The American Society of Hematology.

Mantle cell lymphoma represents approximately 7% of all non-Hodgkin lymphomas (NHLs) and is characterized by the translocation t(11;14)(q13;q32) and the overexpression of *CCND1* (Figure 2). From a tumor biology perspective, two molecular subtypes can be defined; the conventional, nodal type, typically characterized by aggressive clinical course and requiring immediate treatment, and the non-nodal, often leukemic type, with more indolent clinical behavior. The standard approach for younger patients is based on immunochemotherapy, which consists of rituximab and CHOP-like or high-dose Ara-C–containing regimens followed by high-dose treatment with autologous stem cell transplantation (ASCT) and rituximab maintenance. Elderly patients are usually treated with rituximab and CHOP (R-CHOP) or R-bendamustine, followed by rituximab. During the last 5 years, several novel agents have been introduced and approved for the treatment of relapsed and refractory MCL; the immunomodulatory agent lenalidomide, the BTK inhibitor ibrutinib, the BCL2 inhibitor venetoclax, and most recently, brexucabtagene autoleucel, a CAR-T cell product. Many other agents are undergoing clinical development in MCL, including newer generations of BTK inhibitors, bispecific T-cell engagers, and antibody drug conjugates.^58^

### European research contributions

During the last 5 years, European co-operative groups have contributed significantly to the development of treatment strategies for the young as well as the elderly MCL population. In the French LyMa trial, rituximab maintenance post ASCT has been shown to prolong overall survival in the first-line setting, a finding now implemented as standard practice.^59^ A few trials have also explored the use of lenalidomide maintenance. In a phase 3 trial by the Italian FIL group, lenalidomide post ASCT was shown to improve PFS,^60^ and in the 2nd European MCL Network Elderly trial, the addition of lenalidomide to rituximab maintenance (R2) prolonged PFS compared with rituximab alone.^61^ European groups have also explored the use of BTK and BCL2 inhibitors in untreated patients with MCL, such as the combination of rituximab and ibrutinib (IR) in low-risk MCL by the Spanish group,^62^ and the addition of venetoclax consolidation after R-BAC^63^ in elderly high-risk MCL. Moreover, a number of novel combinations have undergone study in relapsed/refractory MCL among co-operative groups in Europe, including temsirolimus, an mTOR inhibitor, in combination with R-bendamustine,^64^ ibrutinib-R2,^65^ venetoclax-R2,^66^ as well as obinutuzumab in combination with venetoclax and ibrutinib.^67^

Furthermore, European groups have been instrumental in the identification of the molecular biology and genome and epigenomic alterations of MCL subtypes^68^ and specific molecular high-risk groups of patients, particularly defined by the presence of mutations of *TP53*, but also other genetic aberrations, including *KMT2D* mutations and *CDKN2A* deletions.^69–71^

### Proposed research for the Roadmap

A number of phase 3 trials, with potential to change clinical practice in untreated patients with MCL, are ongoing in Europe. These include the TRIANGLE trial, assessing the impact of addition of ibrutinib in induction and maintenance, as well as challenging the use of ASCT^72^; the European MCL Network Elderly R2 trial, evaluating the addition of cytarabine to standard R-CHOP induction; and the ENRICH trial, comparing a chemo-free regimen, ibrutinib-rituximab, to standard chemoimmunotherapy in elderly patients. Taking this one step further, ibrutinib-rituximab is compared with venetoclax- ibrutinib-rituximab in a randomized phase 2 trial, the OASIS II. Finally, a randomized trial will compare this triple combination to a chemotherapy standard (MCL elderly III).

Other venues of development include ambitions to develop response adapted treatment strategies, based on MRD^66^; incorporation of the new knowledge emerging from biology studies into risk-based strategies, specifically targeting biological high-risk populations such as TP53-mutated MCL; strategies to improve outcome for BTKi-refractory disease; as well as real-world evidence studies, based on the high quality, nation-wide registers present in many European countries.

### Anticipated impact of the research

Mantle cell lymphoma is a rare lymphoma subtype but also a disease where there is an abundance of novel agents with high activity. We expect that incorporation of novel-targeted agents in front-line combinations ultimately will lead to improvement in survival, and possibly even cure, while reducing early and late side effects.

## FOLLICULAR LYMPHOMA

### Introduction

Follicular lymphoma is the second most common lymphoma, with the highest and increasing incidence in Europe (approximately 3.14 cases per 100,000 persons per year).^73^ FL represents a heterogeneous disease both clinically and biologically. The chromosomal translocation t(14;18) and recurrent alterations in epigenetic regulators are pivotal genetic hallmarks. FL primarily affecting older adults, the disease is characterized by a variable clinical course spanning from those patients exhibiting an indolent behavior to high-risk patients with shortened survival such as those who progress within 24 months of initial immunochemotherapy (POD24) or undergo histological transformation. Guidelines for the diagnosis and treatment of FL were outlined recently by the European Society for Medical Oncology.^74^ There is no consensus on standard of care particularly for relapsed disease, but there is increased interest in novel-targeted agents and immunotherapeutic approaches.

### European research contributions

European groups have led the way on both the biological and therapy front.

Major contributions to unraveling the pathogenesis of include better understanding of the premalignant dynamics of FL development, detailed description of the genetic landscape of FL, the contribution of nongenetic determinants such as the microenvironment and the complexity of genetic heterogeneity and evolution. Emerging data demonstrate that mutations present in tumor cells are implicated in phenotypic and functional remodeling of the FL microenvironment, favoring immune escape mechanisms, and providing a favorable niche for FL cell survival. Furthermore, the adoption of single-cell technological approaches illustrate that the continuum of B-cell states are broader than the traditional germinal center cell of origin of FL.

Several new prognostic models including m7-FLIPI,^75^ PRIMA-PI,^76^ and PRIMA 23-gene^77^ have been developed to aid patient risk stratification. European studies have led in the widespread use of immunochemotherapy^78^ and introduction of novel monoclonal antibodies,^79^ resulting in improvement in survival with median overall survival for FL now 15–20 years. More recent trials have focused on chemotherapy-free regimes targeting the FL cells and the tumor microenvironment leading to the first approval of the immunomodulatory agent lenalidomide for the treatment of FL.^80^ Other studies led or with major contributions from European investigators have led to approvals for FL of PI3-kinase inhibitors, tazemetostat, the first epigenetic drug approved in this disease, and chimeric antigen receptor (CAR) T cells.

### Proposed research for the Roadmap

**Figure 3.**
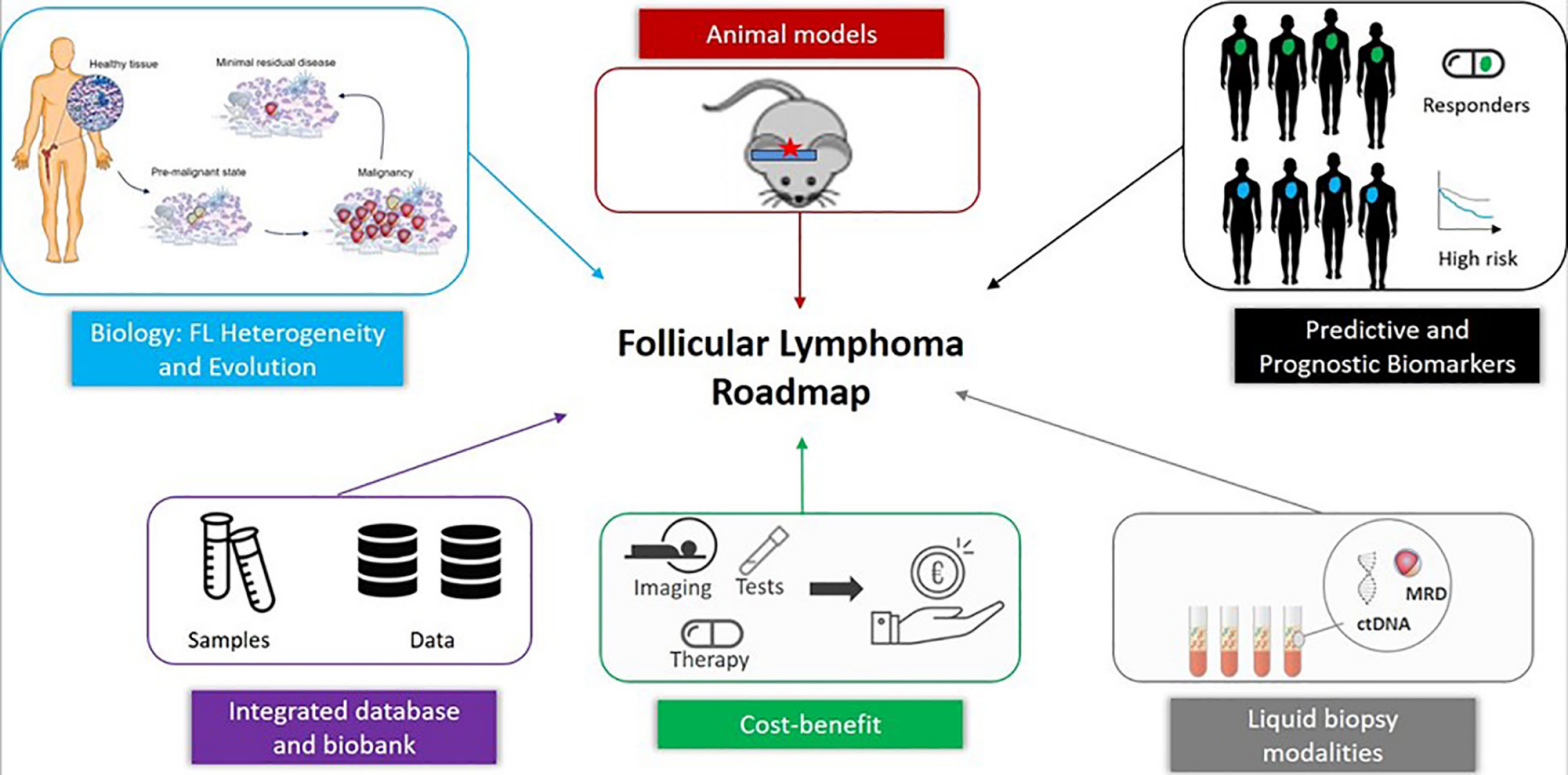
**Research roadmap in FL.** Understanding how healthy B cells transform through premalignancy, to malignancy and how there is persistence of MRD after treatment are key to biologic features; good animal models of FL are key to better understanding pathogenesis and development of novel agents for treatment; development of robust predictive and prognostic biomarkers key to rapid assessment of the utility of new agents; measurement of ctDNA is important to characterize the disease, allow precision medicine approaches and measure MRD; assessing the cost benefit of an integrative approach to treatment of this disease will benefit patients and health technology assessment; integrated database and biobank will provide a pan-European resource to advance study in FL. ctDNA = circulating tumor DNA; FL = follicular lymphoma; MRD = minimal residual disease.

Although major advances have been made in determining the molecular pathogenesis of FL and attempts have been to use this information to develop prognostic scores, it is still not possible to develop scores, which are robust enough or widely applicable to impact clinical management. European researchers are prominent in studies examining the histopathologic changes in the microenvironment and collaborative studies have been performed examining the changes in the tumor microenvironment and how these changes relate to genetic and epigenetic changes in the tumor cells (Figure 3). Europe now leads the way in the use of “big data” to assess the impact of molecular events on outcome but very large cohorts are required to enable all the different parameters to be evaluated. The HARMONY project is now collecting molecular and clinical data from patients with FL, but the number of samples in the database lags behind other disease but this is now being given increased priority.

1. Continue work on the molecular mechanisms spanning early disease development to transformation, should be a common goal.^77,81–85^ Better understanding of the biological underpinnings of different clinical phenotypes and the lymphoma “reservoir,” which likely contribute to subsequent relapse is needed.^83^ A key issue would be performing both genetic and microenvironment analyses on longitudinal or paired FL biopsies to obtain an integrated view on bidirectional dependency.^86,87^
2. A biobank of lymph node and other biopsies linked to the clinical database should be available with protocols for standardized sampling and storage adapted to genomic and functional assays. This requires ethical approval and consent from patients for the procurement and storage of excess tissue from lymph node biopsies at the time of presentation and of particular importance are serial samples obtained from patients in a longitudinal manner at each relapse and progression and there may be value in multiple biopsies to assess intra-patient heterogeneity.^88^
3. A database (a coordinated pan-European registry) that can be accessed by all research partners, containing the biological and clinical information collected for each participating patient, should be made available. This can be provided with the HARMONY platform among others and increased data on FL patients into the database should be encouraged.
4. Robust biomarkers (both prognostic and predictive) should be developed to aid upfront identification of high-risk FL patients and prioritization of specific therapies to specific patient groups.^89^
5. Assays to assess the persistence of MRD should be more widely available and evaluation of emerging “liquid biopsy” and ctDNA assays to provide a means of dynamic risk assessment should be explored.^90,91^
6. Novel animal models that recapitulate disease features and allow preclinical investigation could also be developed. No good animal models of this disease are currently available, limiting research and drug development in this disease.
7. Academic clinical research should address issues related to the costs and benefits of different therapeutic options including the optimal imaging and the potential value of PET scans in this FL into an integrated approach to follow this disease.^91^ This is important to ensure equitable access and affordability to emergent therapies and diagnostic tools.
8. Increased attention should be paid to optimize strategies in the elderly population who carry the major burden of this disease.

### Anticipated impact of the research

The current lack of understanding the nature of the lymphoma “stem cell,” and the events involved in disease progression and transformation negatively impact our ability to cure this disease. The research plans above hold the key to understanding the key molecular events in disease evolution and progression to help identifying key targets for optimal therapeutic intervention, and in particular to target the tumor microenvironment. The characterization of the genetic, genomic, proteomic, transcriptomic, and metabolomic profile of individual patients and patient cohorts and the ability to have these tests widely available for patients in Europe will allow the most appropriate treatment to be selected within clinical trials investigating novel-targeted therapeutic agents. This will also allow identification of robust biomarkers for monitoring response to treatment to allow a precision medicine strategy to be applied to improve the survival and quality of life of patients with FL.

## MARGINAL ZONE LYMPHOMA: EXTRANODAL, NODAL, AND SPLENIC FORMS

### Introduction

Marginal zone lymphomas (MZLs) are a diverse group of clinicοpathological entities, comprising extranodal (also called MALT lymphoma—EMZL), nodal, and splenic (SMZL) forms. Most MZLs are indolent disorders, whose ontogeny is closely linked to autoimmune disorders and chronic infections.^1^ MZLs are often manageable with a “watch and wait” strategy, and, generally, exhibit excellent outcomes when treated with conventional anticancer therapies. That said, open issues exist regarding both the biology and the treatment of MZLs, prompting research that would advance the field toward personalized management.

### European research contributions

An important European milestone concerned the more precise characterization of the molecular landscape of MZL. Next-generation-sequencing studies confirmed previously defined pathways involved in MZ lymphomagenesis (eg, NF-κB and NOTCH), while also highlighting the distinctive features of each entity. Distinct mutation patterns were observed per primary site in EMZL,^92–95^ and distinct mutations in G-protein coupled receptors, not yet reported in human malignancies, have been described.^93^ The immunogenetics of MZLs have been better delineated, revealing distinct biases in different MZLs, strongly supporting unique antigen exposures.^96,97^ Remarkable associations have been reported between particular genomic aberrations and the immunogenic background of MZL, suggesting an intricate cross-talk between the malignant cells and their microenvironment.^98–100^

Several European academic trials shaped the treatment landscape of MZL in the last 5 years. A specific MALT lymphoma prognostic score called “MALT-IPI,” based on the IELSG-19 data set and three further European validation cohorts has been developed.^101^ The negative impact of early progression of disease within 24 months (“POD24”) after start of therapy on survival was confirmed for MALT lymphoma patients.^102^ First-line standards for MALT lymphoma patients in need of systemic treatment have been confirmed by long-term results published for chlorambucil-rituximab (IELSG-19 trial)^103^ and bendamustine-rituximab (MALT 2008-01 trial).^104^ The AUGMENT phase III study has shown that lenalidomide-rituximab was associated with durable responses in MZL patients,^105–107^ and this combination is a salvage option mentioned in the ESMO guidelines.^108^ Encouraging activity has been reported for the second generation anti-CD20-antibody ofatumumab ± bendamustine,^109,110^ and for the small molecules copanlisib and umbralisib.^111^ High-response rates have been reported in SMZL patients treated with rituximab-bendamustine in the BRISMA/IELSG36 trial,^112^ whereas the question on the role of rituximab maintenance in MZL patients remains open.^108,113^ The efficacy of novel antiviral agents for the treatment of hepatitis C virus-triggered MZLs has been suggested by European retrospective studies^114^ and the first prospective trial in this setting, called FIL-BART. Concepts “off-mainstream” are exemplified by data on intralesional rituximab supplemented with autologous serum against MALT lymphoma of the conjunctiva.^115^ Finally, first data of CAR-T-cell therapy for MZL were presented recently; assessment on a larger number of patients and longer follow-up is expected.

### Proposed research for the Roadmap

1. Comprehensive characterization of the biological background of the malignant cells, complemented by functional analysis of their interactions with the respective tumor microenvironments, including micro-organisms.
2. Dissection of disease trajectories and clonal evolution, starting from putative premalignant conditions associated with chronic infections or autoimmunity to entities of undisclosed biological background and uncertain clinical relevance (eg, clonal lymphocytosis of MZ origin) to transformation.
3. Homogenization of antitumor response assessment, especially in view of the particularity of extranodal disease and nonglucose avid lesions at PET, with implications for both routine practice and clinical trials.
4. Identification of ssurrogates of established endpoints in clinical trials, particularly for novel therapeutics, since conventional endpoints, like overall and complete response rates, do not fully capture patient outcomes in MZL.
5. Identification and validation of novel therapeutic approaches aimed at cure, a still unattainable goal, through clinical trials sspecifically focused on MZL patients.

### Anticipated impact of the research

Improved knowledge of MZL biology will result in refined diagnosis and identification of novel druggable targets. Understanding the involved antigens, pathogenic mechanisms, altered molecular pathways, and microenvironmental interactions will promote personalized therapies with better safety and efficacy profiles. Standardization of efficacy endpoints and assessment of surrogate markers will enable reliable comparison between clinical trials. Importantly, only extensive European co-operation will promote the establishment of novel therapeutics in these rare lymphoma entities.

## WALDENSTROM MACROGLOBULINEMIA

### Introduction

WM incidence is usually estimated at around 0.4 per 100,000 person-years, most patients being diagnosed after the age of 65 years. Fixed duration immunochemotherapy (ICT) is widely used since the mid-2000s. Used as first-line therapy, median PFS ranged from around 3 to 5 years. A characteristic mutation in the *MYD88* gene is found in 90–95% of patients. This abnormality pointed out the role of TLR and BCR pathways, among others. A wide range of other molecular abnormalities has also been identified, the most frequent being mutation in the *CXCR4* gene. Meanwhile, ibrutinib, the first-in-class btk inhibitor dramatically improved PFS in refractory/relapsing (RR) patients (4 years median PFS obviously better than previously reported estimates in RR patients).^116^ However, ibrutinib cannot be stopped. The complexity of the genomic landscape suggests that the disease is likely a consequence of a multistep process and that there is a lot of ways to improve outcome with combination therapy (Table 1).

**Table 1.** Summary of Main Steps in the Understanding and the Management of Waldenstrom Macroglobulinemia

Time Scale	Etiology/Epidemiology	Cytogenetic and Genomic	Cytometry/Pathology	Prognosis	Treatment
Present	Preclinical model of mice with MYD88(L252P) mutation^117^	Prospective biobanking		Identification of surrogate endpoint of survival	Fludarabine vs. Chlorambucil^118^
		Optimal method of detection of residual disease?	Optimal threshold and response criteria	Assessment of quality of life	Ibrutinib in previously treated WM patients^119^
				Validation of the prognostic role of molecular landscape	The INNOVATE trial : Rituximab Ibrutinib vs. Rituximab alone^120^
Future	Environmental studies				Prognostic assessment of patients treated with ibrutinib first line
	Preclinical models			Dynamic prediction, New response criteria	Efficacy of vaccines

WM: Waldenstrom macroglobulinemia. Only published original reports or scientific works reported as an abstract since <1 y were considered for this table.

### European research contributions

Swedish teams reported on the frequency of familial WM and on the association with an underlying autoimmune condition, while several Spanish reports deciphered the multistep pattern of onset of asymptomatic WM, and then symptomatic WM as well as the occurrence of histological transformation into an aggressive lymphoma. Furthermore, this group identified a WM-specific phenotype that can be used to assess cellular response in the bone marrow. Two teams of the European consortium established the international scoring system for WM. A revision of this system has been recently proposed by 2 teams of the consortium. European teams also reported on the clinical characteristics of some IgM-related disorders such as neuropathy, acquired von Willebrand syndrome, amyloidosis, and the molecular characteristics of the underlying clonal disorder in the cold agglutinin disease.^116^ Beside guidelines, single centers or single co-operative groups studies, European countries demonstrated their ability to merge their efforts in large-scale multinational studies designed for this rare disease. Following the WM1 trial, the European Consortium for Waldenstrom’s Macroglobulinemia is the largest of its kind and has proven that randomized trial with appropriate power are feasible in this rare disease. An example is the INNOVATE trial, which has led to the approval of Rituximab/Ibrutinib in WM.^120^

### Proposed research for the Roadmap

Prolonged delivery of novel therapy improved the outcome of symptomatic WM patients, compared with the outcome observed after fixed duration immunochemotherapy. The main goal is now to identify a time-limited novel therapy combinations.

### For the present

Systematic adjunction to clinical trials of planned biological studies along with sharing of baseline biological material whenever possible is a major challenge. The availability of this biological information should improve discrimination of currently available scoring systems either for symptomatic or asymptomatic patients. The statistical analyses will have to take into account that one-third of patients with symptomatic WM will not die from the disease. In addition, most molecular abnormalities with potential prognostic significance, including the myd88 wild type status, are found in only small subgroups of patients (<5–10% of patients); the CXCR4 status identified a significant subgroup of patients but the validation of its prognostic value is still pending.^120^

Using the information recorded during the evolution (such as response to treatment) and events that happened meanwhile (such as progression) is another way to improve prognostication. Indeed, current response criteria have a limited prognostic value. Confirmation of the likely prognostic role of flow-cytometry or molecular assessment (including cell-free DNA analyses) of malignant cell depletion in blood or bone marrow is mandatory. In addition, the identification of optimal cutoff values and the integration of this new parameter in new response criteria with validated prognostic values is also a major challenge. This task is probably associated with the definition of surrogate endpoint in place of now unattainable endpoint such as overall survival and/or PFS, especially in the context of first-line therapy.

As already done in other disorders,^121^ it is likely that a large multinational study using specific bioinformatics tools such as artificial intelligence methodology will be required for this purpose. Because of the large number of events and the appropriate follow-up required, this analysis will probably be carried out on patients receiving current treatment approaches, including ICT for most of these, at least at first-line therapy.

Improved baseline prognostic assessment in combination with dynamic tools should improve the design of future trials in the area of new targeted therapy. These tools should also facilitate the identification of particular unmet medical needs and conversely the identification of clinical settings where the mortality may not be different from that of the general population, useful information in case of limited access to health resources.

Finally, during this pandemic, the response to vaccines should also be particularly studied.

All these studies are milestones for improving the quality and the interpretation of efficacy and safety data provided by large multicenter European trials.

The future will rest on a better understanding of the disease, the following issues, among others, remained to be checked: the relationship between the neoplastic B-cell and its microenvironment (Single-cell analyses), the mechanism of clonal evolution (using sequential material). This objective requires a European biobanking network for storing material collected during clinical trials or retrospective studies of interest, once the material is adequately sampled, processed and annotated. In addition, validation of hypotheses provided by these studies will also require appropriate preclinical models.

At any time, it should be reminded of the importance of epidemiological studies for identifying potential environmental risk factors involved in any part of the multistep process of the disease or its clonal evolution. Accurate assessment of the quality of life is also a major endpoint, and there is a need for a specific form specially designed for WM. In Europe, national patient organizations merged their efforts in the European Waldenstrom Network.

## CHRONIC LYMPHOCYTIC LEUKEMIA

### Introduction

CLL is the most common leukemia among adults in Europe.^122,123^ The disease is biologically and clinically complex and can serve as a model system for cancer in general, in particular in the context of senescence and clonal evolution.^124,125^ Based on a better understanding of the disease biology, targeted treatments have been developed that have revolutionized clinical management, yet have led to additional research questions.^126,127^

### European research contributions

European contributions have been of importance with regard to biology as well as therapy of CLL, focusing in particular on the following aspects.

• Molecular mechanisms of pathogenesis, evolution, and resistance

• Biological markers for prognostication, prediction, and as therapeutic targets

• Treatment strategies of improved efficacy and tolerability aiming at cure

### Proposed research for the Roadmap

The cell of origin and initial transforming events of CLL remain largely enigmatic. Monoclonal B-cell lymphocytosis is a condition that appears to precede CLL but the evolution to CLL is rare. Better understanding of the cell of origin and initial transformation steps may lead to diagnostic and therapeutic advances.^128^

The immunoglobulin (Ig) receptor is key in the pathogenesis of the disease, through antigenic or cell-autonomous signaling. The mutational status of the IGHV genes and their stereotyped subsets indicate pathogenic mechanisms, are of prognostic value and can serve for patient stratification. Further research is needed to improve all of these aspects.^129,130^

Ig and other surface receptor biology highlight the importance of the extracellular compartment and the microenvironment. Of note, CLL cells deprived of their in vivo environment die of spontaneous apoptosis. Modeling this interaction of CLL with its environment remains an important issue.^131^

Cell intrinsic abnormalities driving CLL have been defined such as genomics aberrations, gene mutations, methylation abnormalities, and gene expression deregulation. Among these, *TP53* abnormalities and IGHV mutation status/Ig structure are currently the key factors in treatment stratification, but a wealth of additional abnormalities of pathogenic, prognostic, and potentially predictive importance remain to be defined.^125,132^

This is in particular relevant in the light of novel treatment approaches for which prognostic as well as predictive factors need to be established and resistance mechanisms due to the acquired abnormalities of target genes are becoming of importance. Both, hypothesis-driven focused approaches (ie, individual pathways and genes) and global methodologies (ie, “omics” approaches) as well as integration of “big-data,” for example, by machine learning algorithms are needed.^124,125,133^

The importance of Ig and other signaling pathways as well as apoptosis regulation have led to the development of targeted treatments focusing on CD20, BTK, PI3K, and BCL2.^126^ These approaches have revolutionized the treatment of CLL and chemoimmunotherapy plays a minor role only in the absence of *TP53* aberrations and presence of mutated IGHV.^129,132,133^ Among the novel treatment approaches, head-to-head comparisons of single agents and in particular of combinations are a focus of research. Moreover, the best combinations of these agents and the optimal schedule ranging from continuous therapy to fixed duration treatment or response-guided (ie, by MRD) approaches have not been determined and are a priority in clinical trials. Although a comprehensive overview of (European) studies is beyond the scope of this article, many recent trials sponsored by industry (eg, Glow, Sequoia, Captivate, Sequoia, Unity U2) and investigators/study groups (eg, Vision HO141, Filo V+I vs. FCR, UK NCRI FLAIR, GCLLSG CLL13/Gaia, and CLL17) are under way in Europe. This includes also the next-generation agents targeting these pathways whose development is under way. In particular, when comparing fixed duration versus continuous therapy, the sole focus on PFS1 as an endpoint is inadequate and PFS2 as well as OS comparisons after retreatment are needed.

Many patients still experienced disease progression on or after treatment with targeted agents and optimization of sequencing of individual agents or combinations for individual patients defined by host (eg, comorbidities, comedication) and disease (eg, genetics and other biology) characteristics, is a key future question for clinical trials.^127,134^ In disease relapse, a number of resistance mechanisms derived from extracellular but mostly cell intrinsic factors such as mutations of target genes have been identified. The role of T-cell therapy (eg, CARs, bispecific antibodies, stem cell transplantation strategies) in the management of refractory disease merits further investigation.

Apart from CLL progression, transformation into aggressive histology (Richter transformation) is a phenomenon of largely unclear etiology and detrimental outcome.^135^ Prevention and overcoming resistance development, clonal evolution or disease transformation are critical issues for future management.

Although not only efficacy but also tolerability of the targeted agents is favorable, there are adverse events, which are partly characteristic for the novel agents, and need optimization by improved management and further compound development.^136^ In particular, prevention and treatment of infections, and hematological or cardiovascular side effects remain research priorities, as they are leading causes of morbidity and mortality. Because CLL predominates in the elderly, the proportion of older people with CLL will increase and the development of specific tools to assess comorbidities and geriatric status should be pursued. The optimal management of CLL in the COVID-19 era needs international research collaboration.^137^

Overall, it will remain of key importance for future advances to combine laboratory and clinical research in a fully integrated and truly translational approach. This should be reflected in the setup of experimental concepts and clinical trial designs, which must not stand in isolation, with the systematic collection of biological material and information also during long-term follow-up and at progression. From a general perspective, while this outline is focused on CLL, similar principles can be applied to the heterogeneous range of all chronic lymphoproliferative disorders.

### Anticipated impact of the research

Success in laboratory and clinical CLL research has led to improved disease understanding and patient management. Specific impact on CLL is an improved patient outcome, with the overall aim of cure or at least prevention of CLL-related morbidity and mortality. On a larger scale, CLL research can serve as a role model for improved patient management in other conditions, based on the successful translation of disease biology into personalized therapy.

## T-CELL AND NK-CELL LYMPHOMA

### Introduction

Lymphomas arising from mature T- and natural killer (NK)-cells—collectively, peripheral T-cell lymphomas (PTCLs)—are rare, heterogeneous, and poorly understood malignancies, with poor survival outcomes for the majority of patients. The 2017 update of the WHO classification incorporated advances in understanding of the cell of origin and molecular genetics for some entities. However, there is considerable overlap in the genomic landscapes and the existing classification of many PTCLs as “not otherwise specified” (NOS) remains unsatisfactory. Preclinical models remain limited, further hampering development of biologically informed effective therapies. Overall, therapeutic progress has been modest.

### European research contributions

The ECHELON-2 study,^138^ an international effort co-led by European investigators, demonstrated the superiority of CHP plus BV, as compared to CHOP, for patients with Anaplastic Large cell Lymphoma (ALCL). These data led to EMA approval of CHP+BV for ALCL; a paradigm-shift in first-line therapy for this disease. Insights into biological risk stratification in ALCL, including potential prognostic significance of *DUSP22* and *TP63* rearrangements, have not yet impacted clinical decision making. Another large international phase III RCT, led by the LYSA group, reported first data from the Romidepsin (Ro)-CHOP versus CHOP study. However, the primary endpoint was not met; the addition of Romidepsin to CHOP did not improve PFS.^139^ In a phase 2 study (REVAIL) of lenalidomide with CHOP, as first-line treatment for older patients with angioimmunoblastic T-cell lymphoma (AITL), the primary endpoint (complete metabolic response rate) was not met. However, this LYSA study described an association between *DNMT3A* mutations and inferior PFS.^140^ The role of autologous stem cell transplantation (ASCT) for PTCL in first remission remains unresolved.^141^ A German and French co-led study randomised 104 PTCL patients to undergo either ASCT or alloSCT in first remission. No difference in significant 3-year EFS was observed, with relapse representing the dominant cause of failure with auto-SCT, contrasting with no relapses in the alloSCT group but high rates of non-relapse mortality.^142^ For r/r PTCL with a TFH phenotype, the French LYSA group led an international phase 3 study (ORACLE) investigating orally administered 5′-Azacytidine (CC486); results are awaited. As a pivotal proof of concept, an anti-TRBC1 chimeric antigen receptor (CAR) T cell was engineered to recognize and kill TRBC1+, but not TRBC2+, T cells in a preclinical model^143^ paving a path toward adoptive cellular therapy for PTCL. For extranodal NK/T lymphoma, data from the Italian-led International T-cell project demonstrated improved survival outcomes, attributed to widespread adoption of nonanthracycline-based protocols, use of L-asparaginase and radiotherapy.^144^

Investigators from France and Switzerland established that a subset of PTCL-NOS cases display a TFH immunophenotype and similar genetic features to AITL.^145^ Moreover, AITL and related TFH lymphomas harbor diverse recurrent activating mutations in genes related to TCR signaling.^146^ Belgian researchers discovered novel fusions transcripts *FYN-TFNAIP3* and *KDHRSB1* hijacking TCR signaling in PTCL-NOS,^147^ while European researchers contributed to international studies describing novel GEP-defined PTCL subgroups.^148^ A Swiss-led study identified mutation-induced inactivation of the methyltransferase *SETD2* gene as a genomic hallmark of monomorphic epitheliotropic intestinal T-cell lymphoma (MEITL),^149^ with frequent alteration of the same gene in Hepatosplenic T-cell lymphoma (HSTL).^150^ Substantial contributions to the characterization of breast implant-associated ALCLs, were made by European groups: delineating distinct clinical presentations^151^ and new descriptions of the genetic landscape.^152,153^ French researchers developed targeted MLPA-based assays to characterize gene expression signatures of PTCL entities^154^ and to detect known fusion transcripts;^155^ these findings should enable improved characterization of PTCL in routine practice.

### Proposed research for the Roadmap

The overarching research priority is to develop more effective therapies to cure a higher proportion of patients with T and NK lymphomas at first attempt. A deeper understanding of disease pathobiology is necessary to progressively refine diagnosis and prognosis. The molecular complexity of PTCL biology requires a comprehensive understanding of disease processes and tumor heterogeneity. To this end, the European collaborative TRANSCAN-2 project (EuroTCLym) will undertake integrated analysis of PTCL pathobiology on clinically annotated biopsies. Functional studies from representative preclinical models also remain crucial. Clinical trial design must adapt to accommodate multiple biologically defined subgroups of PTCL requiring different therapeutic approaches. Functional imaging has not yet impacted clinical management of PTCL; further focus on advanced imaging metrics is needed. Advances in circulating tumor DNA (ctDNA) technology offers real opportunities both to advance understanding of disease biology through mutational profile analysis and to investigate the utility of ctDNA as a dynamic biomarker. The therapeutic promise of CAR-T cell therapies demands investigation in PTCL. We look toward the very real possibility of rationally designed clinical trials for molecularly defined PTCL subtypes, with therapeutic interventions informed by a refined understanding of specific disease vulnerabilities.

### Anticipated impact of the research

The potential impact of ongoing and planned research programmes in PTCL is substantial. More biologically precise diagnoses, refined prognostication and opportunities for patients to access more effective therapies leading to improved survival, are realistic goals. Nevertheless, significant challenges remain given the rarity of these entities, their biological heterogeneity, and the practical difficulties of enrolling many patients on observational or interventional research protocols. It is particularly crucial for this rare and heterogeneous group of malignancies that pan-European academic and commercial collaborations are broadened and strengthened. Importantly, clinical investigations should remain faithful to the concept of translational science, maximizing the value of data derived from each patient studied, to inform the next generation of clinical research and ultimately improve outcomes for all patients with lymphomas of T- and NK-cell origin.

## LYMPHOMA AND IMMUNE DEFICIENCY (INCLUDING AIDS, POSTTRANSPLANT, AND DRUG-INDUCED IMMUNODEFICIENCY)

### Introduction

The incidence of HL and NHL, in patients with congenital or acquired immune deficiencies, is higher than in the immunocompetent population. AIDS-associated lymphomas are the most representative among them. The incidence of NHL initially fell in the cART era but has now stabilized, whereas that of HL has increased.^156,157^ Posttransplant lymphoproliferative disorders (PTLD) include a range of diseases ranging from benign proliferations to malignant lymphomas.^158^ The management of lymphomas in immunosuppressed patients differs according to the cause of the immunosuppression. In HIV-infected patients, the extensive use of cART has allowed these patients to be treated with identical schedules of immunochemotherapy as those used in the general population (together with cART and adequate prophylaxis of opportunistic infections).^159^ In PTLD, the first step is the removal of immunosuppressive therapy, followed by anti-CD20 immunotherapy, moving quickly to standard immunochemotherapy schedules if response is not rapidly achieved.^160,161^

### European research contributions

Several national groups from European Union countries have conducted phase II trials showing similar results in the treatment of HIV-related lymphomas in the cART era. The most frequent schedules used for DLBCL are R-CHOP, and R-EPOCH (rituximab, etoposide, prednisone, vincristine, cyclophosphamide, and doxorubicin); for BL the most frequent schedules are RCODOX-M/IVAC (rituximab, cyclophosphamide, vincristine, doxorubicin, and methotrexate/ifosfamide, etoposide, and cytarabine), LMB, NHL2002, Burkimab, and dose-adjusted R-EPOCH, among others.^162^ A new prognostic score for HIV-related lymphomas in the rituximab era (AIDS-Related Lymphoma International Prognostic Index) has been developed with important participation of European groups. Similarly, an international effort has been made to define the prognostic factors of HL patients treated with ABVD and cART.

Comparable survival between HIV-positive and HIV-negative NHL and HL patients undergoing autologous peripheral blood stem cell transplantation was observed, leading to the conclusion that, in the cART era, HIV-infected patients with lymphoma should be considered for autologous peripheral blood stem cell transplantation according to the same criteria adopted for HIV-negative lymphoma patients. The same could be applied for PTLD, but special caution should be taken for NRM, primarily driven by infectious toxicity.^163^

### Proposed research for the Roadmap

#### Biological research

(1) To improve the knowledge of the mechanisms of lymphomagenesis in immunosuppression-related lymphomas. (2) To evaluate the potential value of plasma load of gamma herpesviruses as a surrogate marker of residual disease in lymphomas in immunosuppressed patients. (3) To develop early biological predictors of the development of lymphomas in immunosuppressed patients. (4) To study the dynamics of the T-cell and natural killer (NK)-cell repertoire in immunosuppressed patients and its relationship with the development of lymphomas.

#### Clinical research

(1) To develop pan-European clinical trials with the same new drugs used in nonimmunosuppressed patients, especially in the setting of relapsed/refractory status. (2) To develop a joint effort to conduct specific clinical trials for the treatment of infrequent subtypes of lymphoma arising in immunosuppressed patients (eg, plasmablastic, peripheral T-cell, and primary effusion lymphomas). (3) To conduct joint trials with therapies including antiviral agents, adoptive immunotherapy (eg, genetically modified EBV-specific cytotoxic T cells), new drugs, monoclonal antibodies targeting cytokines, and CAR T cells.^164^

### Anticipated impact of the research

The number of lymphomas arising in immunosuppressed patients is expected to increase, making it essential to initiate co-operative efforts to improve the knowledge of the mechanisms of lymphomagenesis and to develop more effective therapies (new drugs and immunologically based therapies), as occurs in nonimmunosuppressed patients. Progress in the knowledge of the mechanisms of lymphoma development in these patients will contribute to improving the treatment results and will hopefully help in the prevention of these lymphomas.

## MULTIPLE MYELOMA AND OTHER PLASMA CELL NEOPLASMS

### European research contributions

#### Minimal residual disease

Bone marrow MRD, by multiparameter flow-cytometry/next-generation flow and NGS, is a valuable long-term outcome predictor in MM.^165^ PET-CT imaging refines determination of MRD negativity.^166^ There is disconcordance between PET-CT and NGS/multiparameter flow-cytometry in approximately 35% of cases.^167^ Therefore, both BM- and imaging-based MRD are now implemented in the MM response criteria.^168^ With current unprecedented lengths of PFS and OS, MRD is a valuable surrogate endpoint for PFS and OS, which would allow earlier registration and access to valuable treatment regimens.

### Next-generation T-cell directing immune therapy

Recently, novel immune therapies, such as anti-BCMA CAR-T cell therapies, improved both PFS and OS in end-stage disease substantially. In addition, the bispecific BCMA-CD3, GPRC5D-CD3, and Fc-RH5-CD3 antibodies are promising approaches with encouraging responses rates.^169–173^

### Patients with unmet medical need

There is still an unmet need for the treatment of patients with high-risk disease based on molecular characteristics and frail patients.^174,175^ It was found that especially patients with a del (17p) clone size of >55% or those with bi-allelic disease harboring a mutation in the *TP53* gene in the other allele have an inferior outcome.^176^ The IMWG frailty score was developed by which the level of frailty can be identified and which is associated with mortality and nonhematologic toxicity.^175^ Thereafter, the first clinical trials were designed for intermediate and frail patients.^177,178^

### Proposed research for the Roadmap

#### Minimal residual disease

Before general introduction of MRD evaluation in daily clinical practice the optimal timing of MRD status has to be defined. Confirming MRD status during therapy is fundamental, because even MRD-negative patients can relapse, and sustained MRD negativity is strongly associated with better outcome.^179^ Furthermore, MRD-driven randomized clinical trials are needed to define the role of therapy intensification in MRD-positive patients or therapy discontinuation in patients with sustained MRD negativity. Several European initiatives are taken.^180^

The value of peripheral blood mass spectrometry measurements should be explored, being more convenient for patients. Preliminary data show concordant results between BM and PB MRD in 80% of patients with a similar prognostic value for PFS. Future studies will have to reveal whether PB MRD can replace BM MRD or is complementary in predicting the outcome.^181^

#### Next-generation T cell directing immune therapy

The optimal timing of CAR-T cell and bispecific antibody therapy needs to be defined. As primary and acquired resistance to T cell directing therapies occur, research on the optimal T-cell repertoire for effective killing and persistence of CAR-T cells is important. In addition, the role of tumor antigen downregulation, the possibilities to increase tumor antigen expression and the value of dual antigen targeting should be investigated. Whether the myeloma immunosuppressive microenvironment can be overcome by the use of immunomodulatory drugs is a research topic of interest both *in vitro,* as well as in clinical trials.^172,182^

Finally, it will be important to define how to choose between CAR-T cell therapy and T-cell directing bispecific antibodies. Will this be guided by tumor or patient characteristics and severity of side effects? Will the current choice based on direct availability be overcome by access to allogeneic off-the-shelf CAR-T cells.^183^ Will there be a possibility to control or cure the disease with CAR-T cell therapy without any additional therapy? Is there a difference in cost-effectiveness?

#### High-risk disease

The current definition of genetic high-risk needs revision. DNA sequencing will allow to detect all copy numbers variations, IGH translocations, and recurrent mutations allowing multiparameter-based more precise risk assessment.^184^ Moreover, it is known that risk evolves over time, therefore, extensive molecular profiling should be performed longitudinally in individual patients to get informed about the clonal evolution and the prognostic impact of the different genetic profiles over time. In addition, it should be investigated whether novel immune therapies will be able to overcome the negative impact of high-risk disease, which has until now only been shown for double autologous transplant.^174,185^ Finally, the development of dedicated high-risk trials is important in order to prevent underpowered sub-analyses of high-risk patients in general randomized clinical trials. Such European initiatives are taken.^186^

#### Frail

There is currently no uniform definition of frailty in clinical practice. Additionally, the discriminative power of current scores is still insufficient to guide treatment decisions. Therefore, further alignment and improvement of the frailty definition is of importance. It would be worthwhile to investigate the impact of functional geriatric assessments. Moreover, dedicated trials for frail patients are urgently needed.^187,188^

#### Anticipated impact of the research

Rational therapy approaches, concerning both the risk-based choice of therapy as well as response- driven modification of therapy, will be enabled by;

(a) refinement of the definition of high-risk molecular disease and frailty improving the prognostic value
(b) addressing the value of novel treatment regimens in high-risk patients by designing separate clinical trials, which will providing more solid data in those patients
(c) increasing knowledge on the predictive value next to the prognostic value of MRD measured by next-generation flow/NGS and PET-CT

Optimization of next-generation immune therapy might lead to a cure for MM given the impressive results in heavily pretreated patients.

The proposed research road map will not only extend but also improve efficacy of the current treatment armamentarium. Importantly, it will allow cost-effective treatment supporting a proper use of health care resources and hopefully decreasing global inequality in drug access due to the rising costs.^189–191^

**Summary box: Main research & policy priorities**

- Establishment and fostering of pan-European scientific networks of both, basic science and clinical lymphoma excellence
- Further elucidation of molecular lymphopathogenesis to identify predictive molecular markers as well as potential targets for future tailored approaches
- Strengthening of academic clinical trials (see also www.bureacracyincts.eu)
- Partnering with patient advocacy groups to incorporate the foremost interests of our patients
- Most importantly, even opportunities for patients all over Europe to receive the optimal current standard of care

## DISCLOSURES

MD receives research support from AbbVie, Bayer, Celgene, Janssen, and Roche; he is a HemaSphere editor; he receives speakers honoraria from Amgen, Astra Zeneca, Bayer, Celgene, Gilead, Janssen, and Roche; and he belongs to the Scientific Advisory Board of Astra Zeneca, Bayer, BMS/Celgene, Beigene, Genmab, Gilead, Incyte, Janssen, Novartis, and Roche. MA is a consultant for Takeda, Bristol-Myers-Squibb, Karyopharm, Gilead, Incyte and receives research support from Roche, Johnson & Johnson, and Takeda. NG receives research support from Amgen, Pfizer, Novartis,Shire/Servier, Jazz Pharmaceuticals, Incyte; she receives honoraria from Amgen, Celgene, Gilead, Novartis, Pfizer, Jazz Pharmaceuticals, Incyte. HT is a member of the advisory board for Roche, Celgene, Incyte. JG provides consultation for Abbvie, Astra Zeneca, BMS, Gilead, Janssen, Morphosys, TG Therapeutics; he receives research support from Astra Zeneca, Janssen and honoraria from Abbvie, BMS, Gilead, Janssen. AF is a member of advisory board for Gilead, Novartis, Roche, Juno, PletixaPharm; he receives research support from BMS, Beigene, Pharmacyclics, Hutchison Medipharma, Amgen, Genmab, ADC Therapeutics, Roche, Gilead, Novartis, Pfizer; he receives honoraria from Adienne and Gilead. SS receives consulting fees, research support and honoraria from AbbVie, Amgen, AstraZeneca, BeiGene, BMS, Celgene, Gilead, GSK, Hoffmann-La Roche, Janssen, Novartis, Sunesis. CF receives consulting fees from AbbVie, AstraZeneca, Atarabio,BMS/Celgene, Gilead/Kite, Janssen, Incyte, Roche, Takeda, BeiGene; he receives research support from BeiGene; he receives honoraria from Takeda, Incyte, Roche, Janssen. JMR receives research support from Amgen, Pfizer, Takeda, Incyte, Servier, Novartis; he receives honoraria from Amgen, Pfizer, Incyte, Servier, Novartis. SZ receives research support from Janssen and Takeda. IA receives financial support from Roche, Janssen, Novartis/Sandoz, Takeda, Amgen, Eusapharma, AbbVie, Oktalpharma/Celtrion, Sanofi Genzyme, Teva/Pliva, Swixx/BMS. CB receives payments for lectures from Abbvie, Jannsen, Pfizer, Roche. BB is member of the advisory board, steering committee and expert for AbbVie, Miletyi, Novartis, Roche. CB received honoraria from Roche, Janssen, BeiGene, Gilead, AbbVie, Celltrion, Pfizer, Novartis, Regeneron, Incyte; he received research funds from Roche, Janssen, AbbVie, MSD, Celltrion, Pfizer, Amgen. EC receives consulting fees from Illumina and AbbVie; he receives honoraria from EUSA Pharma, AstraZeneca; he is author on a patent licensed to NanoString Technologies. BC receives honoraria from BMS, Gilead, Astra Zeneca. AD receives consulting fees from Roche, Acerta/AstraZeneca; he receives research support from Roche, Celgene/BMS, Kite/Gilead, Incyte; he is advisory board member in Roche, Incyte, AstraZeneca, Genmab, Abbvie. FG receives consulting fees from Janssen, Amgen BMS/Celgene, Sanofi, Pfizer, Oncopeptides, GSK, Roche, AbbVie; she receives honoraria from Janssen, Amgen, BMS/Celgene, Sanofi, GSK, AbbVie. PG receives consulting fees from AbbVie, AstraZeneca, ArQule/MSD, BeiGene, Celgene/Juno/BMS, Janssen, Lilly, MEI, Roche, Sanofi; he receives research support from AbbVie, AstraZeneca, Janssen, Sunesis; he is an editor for HemaSphere. HG receives consulting fees from Amgen, BMS, Celgene, Chugai, Janssen, Sanofi; he receives research support from Amgen, BMS, Celgene, Chugai, Janssen, Incyte, Molecular Partners, Merck Sharp and Dohme (MSD), Sanofi, Mundipharma GmbH, Takeda, Novartis; he is an advisory board member for Adaptive Biotechnology, Amgen, BMS, Celgene, Janssen, Sanofi, Takeda; he receives honoraria for Amgen, BMS, Celgene, Chugai, GlaxoSmithKline (GSK), Janssen, Novartis, Sanofi. MJ receives research support from Abbvie, AstraZeneca, Janssen, Roche; he receives honoraria from Janssen, Abbvie, Genmab, Incyte. MJK received research support from Gilead; she receives honoraria from Kite/Gilead, BMS/Celgene, Novartis, Miltenyi Biotec, Roche, Takeda. BK receives honoraria for lectures from AAA, Ipsen, Novartis, MSD, Lilly and is an advisory board member for Ipsen. GL receives consulting fees from Roche, Gilead, Janssen, BMS, Novartis, AstraZeneca, Abbvie, Incyte, MorphoSys, Genmab, Karyopharm, Constellation; he receives research support from Roche, Gilead, Janssen, Bayer, AstraZeneca, MorphoSys; he receives honoraria from Roche, Gilead, Janssen, BMS, Novarts, AstraZeneca, Abbvie, Incyte, MorphoSys. SL receives consulting fees from Genmab, Incyte, Gilead, Orion, CHO Pharma USA, Novartis, Roche; she receives honoraria from Novartis, GILEAD, Incyte. ALG receives consulting fees from Roche, Gilead/Kite, Celgene, Novartis, Astra Zeneca, Abbie, Morphosis, Takeda; he receives research support from Roche, Gilead/Kite, Celgene; he receives honoraria from Roche, Gilead/Kite. MVMM receives honoraria derived from lectures and participation in advisory boards from Janssen, Celgene, Takeda, Amgen, GSK, Abbvie, Pfizer, Regeneron, Adaptive, Roche, Seattle Genentech. PM receives honoraria and is an advisory board member in Celgene/BMS, Janssen, Amgen, Sanofi, AbbVie. CM receives consulting fees from Abbvie, Janssen, AstraZeneca, Beigene; she receives research support from Abbvie and Janssen. JO receives research support from Gilead Science and BeiGene; she receives honoraria from Gilead Science. RO receives honoraria and is an advisory board member in BeiGene and Janssen. TR receives consulting fees from BeiGene, AstraZeneca, Janssen, Abbvie; he receives research support from BeiGene, AstraZeneza, Janssen, Octapharma, Moderna, GSK, Abbvie. KS receives research support from Janssen, Abbvie, Roche, AstraZeneca; he receives honoraria from Janssen, Abbvie, Roche, AstraZeneca. AT receives honoraria and is an advisory board member for Janssen Spa, Beigene, AstraZeneca, AbbVie. RUT receives payments for consultation and attending advisory board meetings from Atara. All the other authors have no conflicts of interest to disclose.

## References

[1] Engert A Balduini C Brand A. The European Hematology Association Roadmap for European Hematology Research: a consensus document. Haematologica. 2016;101:115–208. 26819058 10.3324/haematol.2015.136739 PMC4938336

[2] Sant M Allemani C Tereanu C. Incidence of hematologic malignancies in Europe by morphologic subtype: results of the HAEMACARE project. Blood. 2010;116:3724–3734. 20664057 10.1182/blood-2010-05-282632

[3] Marcos-Gragera R Allemani C Tereanu C. Survival of European patients diagnosed with lymphoid neoplasms in 2000-2002: results of the HAEMACARE project. Haematologica. 2011;96:720–728. 21330324 10.3324/haematol.2010.034264 PMC3084919

[4] Testoni M Zucca E Young KH. Genetic lesions in diffuse large B-cell lymphomas. Ann Oncol. 2015;26:1069–1080. 25605746 10.1093/annonc/mdv019 PMC4542576

[5] Pileri SA Piccaluga PP. New molecular insights into peripheral T cell lymphomas. J Clin Invest. 2012;122:3448–3455. 23023716 10.1172/JCI61205 PMC3461903

[6] Puente XS Pinyol M Quesada V. Whole-genome sequencing identifies recurrent mutations in chronic lymphocytic leukaemia. Nature. 2011;475:101–105. 21642962 10.1038/nature10113 PMC3322590

[7] US National Cancer Institute. International Lymphoma Epidemiology Consortium (InterLymph). 2021. https://epi.grants.cancer.gov/interlymph/. Accessed April 19, 2022. 10.1093/jncimonographs/lgu005 PMC4155460 25174022

[8] Beà S Valdés-Mas R Navarro A. Landscape of somatic mutations and clonal evolution in mantle cell lymphoma. Proc Natl Acad Sci U S A. 2013;110:18250–18255. 24145436 10.1073/pnas.1314608110 PMC3831489

[9] Lemonnier F Couronné L Parrens M. Recurrent TET2 mutations in peripheral T-cell lymphomas correlate with TFH-like features and adverse clinical parameters. Blood. 2012;120:1466–1469. 22760778 10.1182/blood-2012-02-408542

[10] Scott DW Gascoyne RD. The tumour microenvironment in B cell lymphomas. Nat Rev Cancer. 2014;14:517–534. 25008267 10.1038/nrc3774

[11] Chubb D Weinhold N Broderick P. Common variation at 3q26.2, 6p21.33, 17p11.2 and 22q13.1 influences multiple myeloma risk. Nat Genet. 2013;45:1221–1225. 23955597 10.1038/ng.2733 PMC5053356

[12] Cerhan JR Berndt SI Vijai J. Genome-wide association study identifies multiple susceptibility loci for diffuse large B cell lymphoma. Nat Genet. 2014;46:1233–1238. 25261932 10.1038/ng.3105 PMC4213349

[13] Sant M Minicozzi P Mounier M. Survival for haematological malignancies in Europe between 1997 and 2008 by region and age: results of EUROCARE-5, a population-based study. Lancet Oncol. 2014;15:931–942. 25030467 10.1016/S1470-2045(14)70282-7

[14] Hanly P Soerjomataram I Sharp L. Measuring the societal burden of cancer: the cost of lost productivity due to premature cancer-related mortality in Europe. Int J Cancer. 2015;136:E136–E145. 25066804 10.1002/ijc.29105

[15] Oerlemans S Mols F Nijziel MR. The impact of treatment, socio-demographic and clinical characteristics on health-related quality of life among Hodgkin’s and non-Hodgkin’s lymphoma survivors: a systematic review. Ann Hematol. 2011;90:993–1004. 21670973 10.1007/s00277-011-1274-4 PMC3150657

[16] Gallamini A Hutchings M Rigacci L. Early interim 2-[18F]fluoro-2-deoxy-D-glucose positron emission tomography is prognostically superior to international prognostic score in advanced-stage Hodgkin’s lymphoma: a report from a joint Italian-Danish study. J Clin Oncol. 2007;25:3746–3752. 17646666 10.1200/JCO.2007.11.6525

[17] Radford J Illidge T Counsell N. Results of a trial of PET-directed therapy for early-stage Hodgkin’s lymphoma. N Engl J Med. 2015;372:1598–1607. 25901426 10.1056/NEJMoa1408648

[18] André MPE Girinsky T Federico M. Early positron emission tomography response-adapted treatment in stage I and II Hodgkin lymphoma: final results of the randomized EORTC/LYSA/FIL H10 trial. J Clin Oncol. 2017;35:1786–1794. 28291393 10.1200/JCO.2016.68.6394

[19] Fuchs M Goergen H Kobe C. Positron emission tomography-guided treatment in early-stage favorable Hodgkin lymphoma: final results of the International, randomized phase III HD16 trial by the German Hodgkin study Group. J Clin Oncol. 2019;37:2835–2845. 31498753 10.1200/JCO.19.00964

[20] Borchmann P Plütschow A Kobe C. PET-guided omission of radiotherapy in early-stage unfavourable Hodgkin lymphoma (GHSG HD17): a multicentre, open-label, randomised, phase 3 trial. Lancet Oncol. 2021;22:223–234. 33539742 10.1016/S1470-2045(20)30601-X

[21] Johnson P Federico M Kirkwood A. Adapted Treatment Guided by Interim PET-CT Scan in Advanced Hodgkin’s Lymphoma. N Engl J Med. 2016;374:2419–2429. 27332902 10.1056/NEJMoa1510093 PMC4961236

[22] Casasnovas RO Bouabdallah R Brice P. PET-guided, BEACOPPescalated therapy in advanced Hodgkin lymphoma—Authors’ reply. Lancet Oncol. 2019;20:e190. 30942177 10.1016/S1470-2045(19)30156-1

[23] Borchmann P Haverkamp H Lohri A. Progression-free survival of early interim PET-positive patients with advanced stage Hodgkin’s lymphoma treated with BEACOPPescalated alone or in combination with rituximab (HD18): an open-label, international, randomised phase 3 study by the German Hodgkin Study Group. Lancet Oncol. 2017;18:454–463. 28236583 10.1016/S1470-2045(17)30103-1

[24] Mauz-Körholz C Landman-Parker J Balwierz W. Response-adapted omission of radiotherapy and comparison of consolidation chemotherapy in children and adolescents with intermediate-stage and advanced-stage classical Hodgkin lymphoma (EuroNet-PHL-C1): a titration study with an open-label, embedded, multinational, non-inferiority, randomised controlled trial. Lancet Oncol. 2022;23:125–137. 34895479 10.1016/S1470-2045(21)00470-8 PMC8716340

[25] Bröckelmann PJ Goergen H Keller U. Efficacy of Nivolumab and AVD in early-stage unfavorable classic Hodgkin lymphoma: the randomized phase 2 German Hodgkin study group NIVAHL trial. JAMA Oncol. 2020;6:872–880. 32352505 10.1001/jamaoncol.2020.0750 PMC7193521

[26] Fornecker L-M Lazarovici J Aurer I. PET-based response after 2 cycles of brentuximab vedotin in combination with AVD for first-line treatment of unfavorable early-stage Hodgkin lymphoma: first analysis of the primary endpoint of BREACH, a randomized phase 2 trial of LYSA-FIL-EORTC Intergroup. Blood. 2017;130(suppl 1):Abstract 736.

[27] Schmiegelow K Attarbaschi A Barzilai S. Consensus definitions of 14 severe acute toxic effects for childhood lymphoblastic leukaemia treatment: a Delphi consensus. Lancet Oncol. 2016;17:e231–e239. 27299279 10.1016/S1470-2045(16)30035-3

[28] Buchmann S Schrappe M Baruchel A. Remission, treatment failure, and relapse in pediatric ALL: an international consensus of the Ponte-di-Legno Consortium. Blood. 2022;139:1785–1793. 34192312 10.1182/blood.2021012328 PMC8952186

[29] Locatelli F Zugmaier G Rizzari C. Effect of Blinatumomab vs Chemotherapy on event-free survival among children with high-risk first-relapse B-cell acute lymphoblastic leukemia: a randomized clinical trial. JAMA. 2021;325:843–854. 33651091 10.1001/jama.2021.0987 PMC7926287

[30] Gökbuget N Dombret H Bonifacio M. Blinatumomab for minimal residual disease in adults with B-cell precursor acute lymphoblastic leukemia. Blood. 2018;131:1522–1531. 29358182 10.1182/blood-2017-08-798322 PMC6027091

[31] den Boer ML Cario G Moorman AV. Outcomes of paediatric patients with B-cell acute lymphocytic leukaemia with ABL-class fusion in the pre-tyrosine-kinase inhibitor era: a multicentre, retrospective, cohort study. Lancet Haematol. 2021;8:e55–e66. 33357483 10.1016/S2352-3026(20)30353-7 PMC9709453

[32] Peters C Dalle JH Locatelli F. Total body irradiation or chemotherapy conditioning in childhood ALL: a multinational, randomized, noninferiority phase III study. J Clin Oncol. 2021;39:295–307. 33332189 10.1200/JCO.20.02529 PMC8078415

[33] Frismantas V Dobay MP Rinaldi A. Ex vivo drug response profiling detects recurrent sensitivity patterns in drug-resistant acute lymphoblastic leukemia. Blood. 2017;129:e26–e37. 28122742 10.1182/blood-2016-09-738070 PMC5356455

[34] Touzart A Mayakonda A Smith C. Epigenetic analysis of patients with T-ALL identifies poor outcomes and a hypomethylating agent-responsive subgroup. Sci Transl Med. 2021;13:eabc4834. 34039737 10.1126/scitranslmed.abc4834

[35] Enshaei A O’Connor D Bartram J. A validated novel continuous prognostic index to deliver stratified medicine in pediatric acute lymphoblastic leukemia. Blood. 2020;135:1438–1446. 32315382 10.1182/blood.2019003191

[36] Kuhlen M Kunstreich M Gökbuget N. Osteonecrosis in adults with acute lymphoblastic leukemia: an unmet clinical need. Hemasphere. 2021;5:e544. 33718802 10.1097/HS9.0000000000000544 PMC7951118

[37] Gökbuget N Dombret H Ribera JM. International reference analysis of outcomes in adults with B-precursor Ph-negative relapsed/refractory acute lymphoblastic leukemia. Haematologica. 2016;101:1524–1533. 27587380 10.3324/haematol.2016.144311 PMC5479605

[38] Gökbuget N Dombret H Giebel S. Minimal residual disease level predicts outcome in adults with Ph-negative B-precursor acute lymphoblastic leukemia. Hematology. 2019;24:337–348. 30757960 10.1080/16078454.2019.1567654

[39] Locatelli F Whitlock JA Peters C. Blinatumomab versus historical standard therapy in pediatric patients with relapsed/refractory Ph-negative B-cell precursor acute lymphoblastic leukemia. Leukemia. 2020;34:2473–2478. 32094465 10.1038/s41375-020-0770-8 PMC7449874

[40] Foà R Bassan R Vitale A. Dasatinib-blinatumomab for Ph-positive acute lymphoblastic leukemia in adults. N Engl J Med. 2020;383:1613–1623. 33085860 10.1056/NEJMoa2016272

[41] Pocock R Farah N Richardson SE. Current and emerging therapeutic approaches for T-cell acute lymphoblastic leukaemia. Br J Haematol. 2021;194:28–43. 33942287 10.1111/bjh.17310

[42] Wright GW Huang DW Phelan JD. A probabilistic classification tool for genetic subtypes of diffuse large B cell lymphoma with therapeutic implications. Cancer Cell. 2020;37:551–568.e14. 32289277 10.1016/j.ccell.2020.03.015 PMC8459709

[43] Sha C Barrans S Cucco F. Molecular high-grade B-cell lymphoma: defining a poor-risk group that requires different approaches to therapy. J Clin Oncol. 2019;37:202–212. 30523719 10.1200/JCO.18.01314 PMC6338391

[44] Chapuy B Stewart C Dunford AJ. Molecular subtypes of diffuse large B cell lymphoma are associated with distinct pathogenic mechanisms and outcomes. Nat Med. 2018;24:679–690. 29713087 10.1038/s41591-018-0016-8 PMC6613387

[45] Vitolo U Trněný M Belada D. Obinutuzumab or rituximab plus cyclophosphamide, doxorubicin, vincristine, and prednisone in previously untreated diffuse large B-cell lymphoma. J Clin Oncol. 2017;35:3529–3537. 28796588 10.1200/JCO.2017.73.3402

[46] Davies A Cummin TE Barrans S. Gene-expression profiling of bortezomib added to standard chemoimmunotherapy for diffuse large B-cell lymphoma (REMoDL-B): an open-label, randomised, phase 3 trial. Lancet Oncol. 2019;20:649–662. 30948276 10.1016/S1470-2045(18)30935-5 PMC6494978

[47] Thieblemont C Tilly H Gomes da Silva M. Lenalidomide maintenance compared with placebo in responding elderly patients with diffuse large B-cell lymphoma treated with first-line rituximab plus cyclophosphamide, doxorubicin, vincristine, and prednisone. J Clin Oncol. 2017;35:2473–2481. 28426350 10.1200/JCO.2017.72.6984

[48] Poeschel V Held G Ziepert M. Four versus six cycles of CHOP chemotherapy in combination with six applications of rituximab in patients with aggressive B-cell lymphoma with favourable prognosis (FLYER): a randomised, phase 3, non-inferiority trial. Lancet. 2019;394:2271–2281. 31868632 10.1016/S0140-6736(19)33008-9

[49] Dührsen U Müller S Hertenstein B. Positron emission tomography-guided therapy of aggressive Non-Hodgkin lymphomas (PETAL): a multicenter, randomized phase III trial. J Clin Oncol. 2018;36:2024–2034. 29750632 10.1200/JCO.2017.76.8093

[50] Le Gouill S Ghesquières H Oberic L. Obinutuzumab vs rituximab for advanced DLBCL: a PET-guided and randomized phase 3 study by LYSA. Blood. 2021;137:2307–2320. 33211799 10.1182/blood.2020008750

[51] Hutchings M Morschhauser F Iacoboni G. Glofitamab, a Novel, Bivalent CD20-Targeting T-Cell-Engaging Bispecific Antibody, Induces Durable Complete Remissions in Relapsed or Refractory B-Cell Lymphoma: a Phase I Trial. J Clin Oncol. 2021;39:1959–1970. 33739857 10.1200/JCO.20.03175 PMC8210975

[52] Salles G Duell J González Barca E. Tafasitamab plus lenalidomide in relapsed or refractory diffuse large B-cell lymphoma (L-MIND): a multicentre, prospective, single-arm, phase 2 study. Lancet Oncol. 2020;21:978–988. 32511983 10.1016/S1470-2045(20)30225-4

[53] Morschhauser F Flinn IW Advani R. Polatuzumab vedotin or pinatuzumab vedotin plus rituximab in patients with relapsed or refractory non-Hodgkin lymphoma: final results from a phase 2 randomised study (ROMULUS). Lancet Haematol. 2019;6:e254–e265. 30935953 10.1016/S2352-3026(19)30026-2

[54] Tilly H Morschhauser F Bartlett NL. Polatuzumab vedotin in combination with immunochemotherapy in patients with previously untreated diffuse large B-cell lymphoma: an open-label, non-randomised, phase 1b-2 study. Lancet Oncol. 2019;20:998–1010. 31101489 10.1016/S1470-2045(19)30091-9

[55] Caimi PF Ai W Alderuccio JP. Loncastuximab tesirine in relapsed or refractory diffuse large B-cell lymphoma (LOTIS-2): a multicentre, open-label, single-arm, phase 2 trial. Lancet Oncol. 2021;22:790–800. 33989558 10.1016/S1470-2045(21)00139-X

[56] Minard-Colin V Aupérin A Pillon M. Rituximab for high-risk, mature B-cell Non-Hodgkin’s lymphoma in children. N Engl J Med. 2020;382:2207–2219. 32492302 10.1056/NEJMoa1915315 PMC7720281

[57] Au-Yeung RKH Arias Padilla L Zimmermann M. Experience with provisional WHO-entities large B-cell lymphoma with IRF4-rearrangement and Burkitt-like lymphoma with 11q aberration in paediatric patients of the NHL-BFM group. Br J Haematol. 2020;190:753–763. 32239695 10.1111/bjh.16578

[58] Swerdlow SH Campo E Pileri SA. The 2016 revision of the World Health Organization classification of lymphoid neoplasms. Blood. 2016;127:2375–2390. 26980727 10.1182/blood-2016-01-643569 PMC4874220

[59] Le Gouill S Thieblemont C Oberic L. Rituximab after autologous stem-cell transplantation in mantle-cell lymphoma. N Engl J Med. 2017;377:1250–1260. 28953447 10.1056/NEJMoa1701769

[60] Ladetto M Ferrero S Evangelista A. Lenalidomide maintenance after autologous transplantation prolongs PFS in young MCL patients: results of the randomized phase III MCL 0208 trial from Fondazione Italiana Linfomi (FIL). Blood. 2018;132(suppl 1):401.

[61] Ribrag V Safar V Kluin-Nelemans H. Rituximab-Lenalidomide(R2) maintenance is superior to rituximab maintenance after first line immunochemotherapy in mantle cell lymphoma: results of the MCL R2 elderly clinical trial. Blood. 2021;138(suppl 1):379.

[62] Giné E de la Cruz F Jiménez Ubieto A. Ibrutinib in combination with rituximab for indolent clinical forms of mantle cell lymphoma (IMCL-2015): a multicenter, open-label, single-arm, phase II trial. J Clin Oncol. 2022;40:1196–1205. 35030036 10.1200/JCO.21.02321 PMC8987223

[63] Visco C Tabanelli V Peracchio C. Rituximab, bendamustine and cytarabine followed By venetoclax (V-RBAC) in high-risk elderly patients with mantle cell lymphoma. Blood. 2021;138(suppl 1):2427.

[64] Hess G Karola W LaRosee P. Temsirolimus in combination with bendamustine and rituximab (BeRT) for the treatment of relapsed mantle cell and follicular lymphoma: final phase I/II results. Blood. 2016;128:2977.

[65] Jerkeman M Eskelund CW Hutchings M. Ibrutinib, lenalidomide, and rituximab in relapsed or refractory mantle cell lymphoma (PHILEMON): a multicentre, open-label, single-arm, phase 2 trial. Lancet Haematol. 2018;5:e109–e116. 29396091 10.1016/S2352-3026(18)30018-8

[66] Jerkeman M Kolstad A Niemann CU. Venetoclax, lenalidomide and rituximab for patients with relapsed or refractory mantle cell lymphoma—data from the nordic lymphoma group NLG-MCL7 (VALERIA) phase I trial: stopping treatment in molecular remission is feasible. Blood. 2020;136(suppl 1):15.

[67] Le Gouill S Morschhauser F Chiron D. Ibrutinib, obinutuzumab, and venetoclax in relapsed and untreated patients with mantle cell lymphoma: a phase ½ trial. Blood. 2021;137:877–887. 33181832 10.1182/blood.2020008727

[68] Nadeu F Martin-Garcia D Clot G. Genomic and epigenomic insights into the origin, pathogenesis, and clinical behavior of mantle cell lymphoma subtypes. Blood. 2020;136:1419–1432. 32584970 10.1182/blood.2020005289 PMC7498364

[69] Ferrero S Rossi D Rinaldi A. KMT2D mutations and TP53 disruptions are poor prognostic biomarkers in mantle cell lymphoma receiving high-dose therapy: a FIL study. Haematologica. 2020;105:1604–1612. 31537689 10.3324/haematol.2018.214056 PMC7271566

[70] Eskelund CW Dahl C Hansen JW. TP53 mutations identify younger mantle cell lymphoma patients who do not benefit from intensive chemoimmunotherapy. Blood. 2017;130:1903–1910. 28819011 10.1182/blood-2017-04-779736

[71] Malarikova D Berkova A Obr A. Concurrent TP53 and CDKN2A gene aberrations in newly diagnosed mantle cell lymphoma correlate with chemoresistance and call for innovative upfront therapy. Cancers (Basel). 2020;12:E2120. 32751805 10.3390/cancers12082120 PMC7466084

[72] Dreyling M Ladetto M Doorduijn JK. Triangle: autologous transplantation after a Rituximab/Ibrutinib/ara-c containing induction in generalized mantle cell lymphoma—a randomized European MCL Network Trial. Blood. 2019;134(suppl 1):2816.

[73] Smith A Crouch S Lax S. Lymphoma incidence, survival and prevalence 2004-2014: sub-type analyses from the UK’s Haematological Malignancy Research Network. Br J Cancer. 2015;112:1575–1584. 25867256 10.1038/bjc.2015.94 PMC4453686

[74] Dreyling M Ghielmini M Rule S. Newly diagnosed and relapsed follicular lymphoma: ESMO Clinical Practice Guidelines for diagnosis, treatment and follow-up. Ann Oncol. 2021;32:298–308. 33249059 10.1016/j.annonc.2020.11.008

[75] Pastore A Jurinovic V Kridel R. Integration of gene mutations in risk prognostication for patients receiving first-line immunochemotherapy for follicular lymphoma: a retrospective analysis of a prospective clinical trial and validation in a population-based registry. Lancet Oncol. 2015;16:1111–1122. 26256760 10.1016/S1470-2045(15)00169-2

[76] Bachy E Maurer MJ Habermann TM. A simplified scoring system in de novo follicular lymphoma treated initially with immunochemotherapy. Blood. 2018;132:49–58. 29666118 10.1182/blood-2017-11-816405 PMC6034646

[77] Huet S Tesson B Jais JP. A gene-expression profiling score for prediction of outcome in patients with follicular lymphoma: a retrospective training and validation analysis in three international cohorts. Lancet Oncol. 2018;19:549–561. 29475724 10.1016/S1470-2045(18)30102-5 PMC5882539

[78] Bachy E Seymour JF Feugier P. Sustained progression-free survival benefit of rituximab maintenance in patients with follicular lymphoma: long-term results of the PRIMA study. J Clin Oncol. 2019;37:2815–2824. 31339826 10.1200/JCO.19.01073 PMC6823890

[79] Marcus R Davies A Ando K. Obinutuzumab for the first-line treatment of follicular lymphoma. N Engl J Med. 2017;377:1331–1344. 28976863 10.1056/NEJMoa1614598

[80] Leonard JP Trneny M Izutsu K. AUGMENT: a phase III study of lenalidomide plus rituximab versus placebo plus rituximab in relapsed or refractory indolent lymphoma. J Clin Oncol. 2019;37:1188–1199. 30897038 10.1200/JCO.19.00010 PMC7035866

[81] Heward J Konali L D’Avola A. KDM5 inhibition offers a novel therapeutic strategy for the treatment of KMT2D mutant lymphomas. Blood. 2021;138:370–381. 33786580 10.1182/blood.2020008743 PMC8351530

[82] Ortega-Molina A Deleyto-Seldas N Carreras J. Oncogenic Rag GTPase signaling enhances B cell activation and drives follicular lymphoma sensitive to pharmacological inhibition of mTOR. Nat Metab. 2019;1:775–789. 31579886 10.1038/s42255-019-0098-8 PMC6774795

[83] Horton SJ Giotopoulos G Yun H. Early loss of Crebbp confers malignant stem cell properties on lymphoid progenitors. Nat Cell Biol. 2017;19:1093–1104. 28825697 10.1038/ncb3597 PMC5633079

[84] Bararia D Hildebrand JA Stolz S. Cathepsin S alterations induce a tumor-promoting immune microenvironment in follicular lymphoma. Cell Rep. 2020;31:107522. 32330423 10.1016/j.celrep.2020.107522

[85] Milpied P Cervera-Marzal I Mollichella ML. Human germinal center transcriptional programs are de-synchronized in B cell lymphoma. Nat Immunol. 2018;19:1013–1024. 30104629 10.1038/s41590-018-0181-4

[86] Stevens WBC Mendeville M Redd R. Prognostic relevance of CD163 and CD8 combined with EZH2 and gain of chromosome 18 in follicular lymphoma: a study by the Lunenburg Lymphoma Biomarker Consortium. Haematologica. 2017;102:1413–1423. 28411252 10.3324/haematol.2017.165415 PMC6643731

[87] Mourcin F Verdiére L Roulois D. Follicular lymphoma triggers phenotypic and functional remodeling of the human lymphoid stromal cell landscape. Immunity. 2021;54:1901. 34380065 10.1016/j.immuni.2021.07.018

[88] Araf S Wang J Korfi K. Genomic profiling reveals spatial intra-tumor heterogeneity in follicular lymphoma. Leukemia. 2018;32:1261–1265. 29568095 10.1038/s41375-018-0043-y PMC5940637

[89] Mozas P Rivero A López-Guillermo A. Past, present and future of prognostic scores in follicular lymphoma. Blood Rev. 2021;50:100865. 34187710 10.1016/j.blre.2021.100865

[90] Nagy Á Bátai B Balogh A. Quantitative analysis and monitoring of EZH2 mutations using liquid biopsy in follicular lymphoma. Genes (Basel). 2020;11:E785. 32668764 10.3390/genes11070785 PMC7397208

[91] Delfau-Larue MH van der Gucht A Dupuis J. Total metabolic tumor volume, circulating tumor cells, cell-free DNA: distinct prognostic value in follicular lymphoma. Blood Adv. 2018;2:807–816. 29636326 10.1182/bloodadvances.2017015164 PMC5894260

[92] Cascione L Rinaldi A Bruscaggin A. Novel insights into the genetics and epigenetics of MALT lymphoma unveiled by next generation sequencing analyses. Haematologica. 2019;104:e558–e561. 31018978 10.3324/haematol.2018.214957 PMC6959164

[93] Moody S Thompson JS Chuang SS. Novel GPR34 and CCR6 mutation and distinct genetic profiles in MALT lymphomas of different sites. Haematologica. 2018;103:1329–1336. 29674500 10.3324/haematol.2018.191601 PMC6068028

[94] Wu F Watanabe N Tzioni MM. Thyroid MALT lymphoma: self-harm to gain potential T-cell help. Leukemia. 2021;35:3497–3508. 34021249 10.1038/s41375-021-01289-z PMC8632687

[95] Kiesewetter B Copie-Bergman C Levy M. Genetic characterization and clinical features of helicobacter pylori negative gastric mucosa-associated lymphoid tissue lymphoma. Cancers (Basel). 2021;13:2993. 34203889 10.3390/cancers13122993 PMC8232676

[96] Bikos V Karypidou M Stalika E. An immunogenetic signature of ongoing antigen interactions in splenic marginal zone lymphoma expressing IGHV1-2*04 receptors. Clin Cancer Res. 2016;22:2032–2040. 26647217 10.1158/1078-0432.CCR-15-1170

[97] Xochelli A Bikos V Polychronidou E. Disease-biased and shared characteristics of the immunoglobulin gene repertoires in marginal zone B cell lymphoproliferations. J Pathol. 2019;247:416–421. 30484876 10.1002/path.5209

[98] Agathangelidis A Xochelli A Stamatopoulos K. A gene is known by the company it keeps: enrichment of TNFAIP3 gene aberrations in MALT lymphomas expressing IGHV4-34 antigen receptors. J Pathol. 2017;243:403–406. 28892161 10.1002/path.4982

[99] Parry M Rose-Zerilli MJ Ljungström V. Genetics and prognostication in splenic marginal zone lymphoma: revelations from deep sequencing. Clin Cancer Res. 2015;21:4174–4183. 25779943 10.1158/1078-0432.CCR-14-2759 PMC4490180

[100] Moody S Escudero-Ibarz L Wang M. Significant association between TNFAIP3 inactivation and biased immunoglobulin heavy chain variable region 4-34 usage in mucosa-associated lymphoid tissue lymphoma. J Pathol. 2017;243:3–8. 28682481 10.1002/path.4933

[101] Thieblemont C Cascione L Conconi A. A MALT lymphoma prognostic index. Blood. 2017;130:1409–1417. 28720586 10.1182/blood-2017-03-771915

[102] Conconi A Thieblemont C Cascione L. Early progression of disease predicts shorter survival in MALT lymphoma patients receiving systemic treatment. Haematologica. 2020;105:2592–2597. 33131248 10.3324/haematol.2019.237990 PMC7604574

[103] Zucca E Conconi A Martinelli G. Final results of the IELSG-19 randomized trial of mucosa-associated lymphoid tissue lymphoma: improved event-free and progression-free survival with rituximab plus chlorambucil versus either chlorambucil or rituximab monotherapy. J Clin Oncol. 2017;35:1905–1912. 28355112 10.1200/JCO.2016.70.6994

[104] Salar A Domingo-Domenech E Panizo C. Long-term results of a phase 2 study of rituximab and bendamustine for mucosa-associated lymphoid tissue lymphoma. Blood. 2017;130:1772–1774. 28801448 10.1182/blood-2017-07-795302

[105] Kiesewetter B Willenbacher E Willenbacher W. A phase 2 study of rituximab plus lenalidomide for mucosa-associated lymphoid tissue lymphoma. Blood. 2017;129:383–385. 27879257 10.1182/blood-2016-06-720599

[106] Kiesewetter B Lamm W Neuper O. Prolonged follow-up on lenalidomide-based treatment for mucosa-associated lymphoid tissue lymphoma (MALT lymphoma)-Real-world data from the Medical University of Vienna. Hematol Oncol. 2019;37:345–351. 31283840 10.1002/hon.2647 PMC6899635

[107] Becnel MR Nastoupil LJ Samaniego F. Lenalidomide plus rituximab (R2) in previously untreated marginal zone lymphoma: subgroup analysis and long-term follow-up of an open-label phase 2 trial. Br J Haematol. 2019;185:874–882. 30919940 10.1111/bjh.15843 PMC6619290

[108] Zucca E Arcaini L Buske C. Marginal zone lymphomas: ESMO Clinical Practice Guidelines for diagnosis, treatment and follow-up. Ann Oncol. 2020;31:17–29. 31912792 10.1016/j.annonc.2019.10.010

[109] Vannata B Vanazzi A Negri M. A phase II trial of bendamustine in combination with ofatumumab in patients with relapsed or refractory marginal zone B-cell lymphomas. Hematol Oncol. 2021;39:60–65. 33103778 10.1002/hon.2822

[110] Kiesewetter B Neuper O Mayerhoefer ME. A pilot phase II study of ofatumumab monotherapy for extranodal marginal zone B-cell lymphoma of the mucosa-associated lymphoid tissue (MALT) lymphoma. Hematol Oncol. 2018;36:49–55. 28695630 10.1002/hon.2454

[111] Panayiotidis P Follows GA Mollica L. Efficacy and safety of copanlisib in patients with relapsed or refractory marginal zone lymphoma. Blood Adv. 2021;5:823–828. 33560394 10.1182/bloodadvances.2020002910 PMC7876879

[112] Iannitto E Bellei M Amorim S. Efficacy of bendamustine and rituximab in splenic marginal zone lymphoma: results from the phase II BRISMA/IELSG36 study. Br J Haematol. 2018;183:755–765. 30407629 10.1111/bjh.15641

[113] Stathis A Gregorini A Gressin R. IELSG-38: a phase II study of chlorambucil in combination with rituximab followed By maintenance therapy with subcutaneous rituximab in patients with extranodal marginal zone B-cell lymphoma of mucosa associated lymphoid tissue (MALT). Blood. 2017;130(suppl 1):1506.

[114] Frigeni M Besson C Visco C. Interferon-free compared to interferon-based antiviral regimens as first-line therapy for B-cell lymphoproliferative disorders associated with hepatitis C virus infection. Leukemia. 2020;34:1462–1466. 31836855 10.1038/s41375-019-0687-2

[115] Ferreri AJM Sassone M Miserocchi E. Treatment of MALT lymphoma of the conjunctiva with intralesional rituximab supplemented with autologous serum. Blood Adv. 2020;4:1013–1019. 32182364 10.1182/bloodadvances.2020001459 PMC7094013

[116] Leblond V Treon SP Dimopoulos MA. Waldenström’s macroglobulinemia. Switzerland: Springer International Publishing; 2017.

[117] Ouk C Roland L Gachard N. Continuous MYD88 activation is associated with expansion and then transformation of IgM differentiating plasma cells. Front Immunol. 2021;12:641692. 34017329 10.3389/fimmu.2021.641692 PMC8129569

[118] Leblond V Johnson S Chevret S. Results of a randomized trial of chlorambucil versus fludarabine for patients with untreated Waldenström macroglobulinemia, marginal zone lymphoma, or lymphoplasmacytic lymphoma. J Clin Oncol. 2013;31:301–307. 23233721 10.1200/JCO.2012.44.7920

[119] Treon SP Tripsas CK Meid K. Ibrutinib in previously treated Waldenström’s macroglobulinemia. N Engl J Med. 2015;372:1430–1440. 25853747 10.1056/NEJMoa1501548

[120] Dimopoulos MA Tedeschi A Trotman J. Phase 3 trial of Ibrutinib plus rituximab in Waldenström’s macroglobulinemia. N Engl J Med. 2018;378:2399–2410. 29856685 10.1056/NEJMoa1802917

[121] Kurtz DM Esfahani MS Scherer F. Dynamic risk profiling using serial tumor biomarkers for personalized outcome prediction. Cell. 2019;178:699–713.e19. 31280963 10.1016/j.cell.2019.06.011 PMC7380118

[122] Eichhorst B Robak T Montserrat E. Chronic lymphocytic leukaemia: ESMO Clinical Practice Guidelines for diagnosis, treatment and follow-up. Ann Oncol. 2021;32:23–33. 33091559 10.1016/j.annonc.2020.09.019

[123] Hallek M Cheson BD Catovsky D. iwCLL guidelines for diagnosis, indications for treatment, response assessment, and supportive management of CLL. Blood. 2018;131:2745–2760. 29540348 10.1182/blood-2017-09-806398

[124] Zapatka M Tausch E Öztürk S. Clonal evolution in chronic lymphocytic leukemia is scant in relapsed but accelerated in refractory cases after chemo(immune) therapy. Haematologica. 2022;107:604–614. 33691380 10.3324/haematol.2020.265777 PMC8883533

[125] Knisbacher B Lin Z Hahn CK. Molecular map of chronic lymphocytic leukemia and its impact on outcome. Nat Genet. 2022. [In Press]. 10.1038/s41588-022-01140-w PMC10084830 35927489

[126] Al-Sawaf O Zhang C Tandon M. Venetoclax plus obinutuzumab versus chlorambucil plus obinutuzumab for previously untreated chronic lymphocytic leukaemia (CLL14): follow-up results from a multicentre, open-label, randomised, phase 3 trial. Lancet Oncol. 2020;21:1188–1200. 32888452 10.1016/S1470-2045(20)30443-5

[127] International CLL-IPI working group. An international prognostic index for patients with chronic lymphocytic leukaemia (CLL-IPI): a meta-analysis of individual patient data. Lancet Oncol. 2016;17:779–790. 27185642 10.1016/S1470-2045(16)30029-8

[128] Rawstron AC Kreuzer KA Soosapilla A. Reproducible diagnosis of chronic lymphocytic leukemia by flow cytometry: an European Research Initiative on CLL (ERIC) & European Society for Clinical Cell Analysis (ESCCA) Harmonisation project. Cytometry B Clin Cytom. 2018;94:121–128. 29024461 10.1002/cyto.b.21595 PMC5817234

[129] Rosenquist R Ghia P Hadzidimitriou A. Immunoglobulin gene sequence analysis in chronic lymphocytic leukemia: updated ERIC recommendations. Leukemia. 2017;31:1477–1481. 28439111 10.1038/leu.2017.125 PMC5508071

[130] Agathangelidis A Chatzidimitriou A Gemenetzi K. Higher-order connections between stereotyped subsets: implications for improved patient classification in CLL. Blood. 2021;137:1365–1376. 32992344 10.1182/blood.2020007039 PMC7976441

[131] Minici C Gounari M Übelhart R. Distinct homotypic B-cell receptor interactions shape the outcome of chronic lymphocytic leukaemia. Nat Commun. 2017;8:15746. 28598442 10.1038/ncomms15746 PMC5472768

[132] Malcikova J Tausch E Rossi D. ERIC recommendations for TP53 mutation analysis in chronic lymphocytic leukemia-update on methodological approaches and results interpretation. Leukemia. 2018;32:1070–1080. 29467486 10.1038/s41375-017-0007-7 PMC5940638

[133] Baliakas P Jeromin S Iskas M. Cytogenetic complexity in chronic lymphocytic leukemia: definitions, associations, and clinical impact. Blood. 2019;133:1205–1216. 30602617 10.1182/blood-2018-09-873083 PMC6509568

[134] Tausch E Schneider C Robrecht S. Prognostic and predictive impact of genetic markers in patients with CLL treated with obinutuzumab and venetoclax. Blood. 2020;135:2402–2412. 32206772 10.1182/blood.2019004492

[135] Condoluci A Rossi D. Richter Syndrome. Curr Oncol Rep. 2021;23:26. 33580422 10.1007/s11912-020-01001-x PMC7880969

[136] Gribben JG Bosch F Cymbalista F. Optimising outcomes for patients with chronic lymphocytic leukaemia on ibrutinib therapy: European recommendations for clinical practice. Br J Haematol. 2018;180:666–679. 29318593 10.1111/bjh.15080

[137] Scarfò L Chatzikonstantinou T Rigolin GM. COVID-19 severity and mortality in patients with chronic lymphocytic leukemia: a joint study by ERIC, the European Research Initiative on CLL, and CLL Campus. Leukemia. 2020;34:2354–2363. 32647324 10.1038/s41375-020-0959-x PMC7347048

[138] Horwitz S O’Connor OA Pro B. Brentuximab vedotin with chemotherapy for CD30-positive peripheral T-cell lymphoma (ECHELON-2): a global, double-blind, randomised, phase 3 trial. Lancet. 2019;393:229–240. 30522922 10.1016/S0140-6736(18)32984-2 PMC6436818

[139] Bachy E Camus V Thieblemont C. Final analysis of the Ro-CHOP phase III study (Conducted by LYSA): romidepsin plus CHOP in patients with peripheral T-cell lymphoma. Blood. 2020;136(suppl 1):32–33.

[140] Lemonnier F Safar V Beldi-Ferchiou A. Integrative analysis of a phase 2 trial combining lenalidomide with CHOP in angioimmunoblastic T-cell lymphoma. Blood Adv. 2021;5:539–548. 33496747 10.1182/bloodadvances.2020003081 PMC7839364

[141] Fossard G Broussais F Coelho I. Role of up-front autologous stem-cell transplantation in peripheral T-cell lymphoma for patients in response after induction: an analysis of patients from LYSA centers. Ann Oncol. 2018;29:715–723. 29253087 10.1093/annonc/mdx787

[142] Schmitz N Truemper L Bouabdallah K. A randomized phase 3 trial of autologous vs allogeneic transplantation as part of first-line therapy in poor-risk peripheral T-NHL. Blood. 2021;137:2646–2656. 33512419 10.1182/blood.2020008825 PMC9635528

[143] Maciocia PM Wawrzyniecka PA Philip B. Targeting the T cell receptor β-chain constant region for immunotherapy of T cell malignancies. Nat Med. 2017;23:1416–1423. 29131157 10.1038/nm.4444

[144] Fox CP Civallero M Ko YH. Survival outcomes of patients with extranodal natural-killer T-cell lymphoma: a prospective cohort study from the international T-cell Project. Lancet Haematol. 2020;7:e284–e294. 32105608 10.1016/S2352-3026(19)30283-2

[145] Dobay MP Lemonnier F Missiaglia E. Integrative clinicopathological and molecular analyses of angioimmunoblastic T-cell lymphoma and other nodal lymphomas of follicular helper T-cell origin. Haematologica. 2017;102:e148–e151. 28082343 10.3324/haematol.2016.158428 PMC5395128

[146] Vallois D Dobay MP Morin RD. Activating mutations in genes related to TCR signaling in angioimmunoblastic and other follicular helper T-cell-derived lymphomas. Blood. 2016;128:1490–1502. 27369867 10.1182/blood-2016-02-698977

[147] Debackere K Marcelis L Demeyer S. Fusion transcripts FYN-TRAF3IP2 and KHDRBS1-LCK hijack T cell receptor signaling in peripheral T-cell lymphoma, not otherwise specified. Nat Commun. 2021;12:3705. 34140493 10.1038/s41467-021-24037-4 PMC8211700

[148] Heavican TB Bouska A Yu J. Genetic drivers of oncogenic pathways in molecular subgroups of peripheral T-cell lymphoma. Blood. 2019;133:1664–1676. 30782609 10.1182/blood-2018-09-872549 PMC6460420

[149] Roberti A Dobay MP Bisig B. Type II enteropathy-associated T-cell lymphoma features a unique genomic profile with highly recurrent SETD2 alterations. Nat Commun. 2016;7:12602. 27600764 10.1038/ncomms12602 PMC5023950

[150] McKinney M Moffitt AB Gaulard P. The genetic basis of hepatosplenic T-cell lymphoma. Cancer Discov. 2017;7:369–379. 28122867 10.1158/2159-8290.CD-16-0330 PMC5402251

[151] Laurent C Delas A Gaulard P. Breast implant-associated anaplastic large cell lymphoma: two distinct clinicopathological variants with different outcomes. Ann Oncol. 2016;27:306–314. 26598546 10.1093/annonc/mdv575 PMC4722894

[152] Laurent C Nicolae A Laurent C. Gene alterations in epigenetic modifiers and JAK-STAT signaling are frequent in breast implant-associated ALCL. Blood. 2020;135:360–370. 31774495 10.1182/blood.2019001904 PMC7059458

[153] de Leval L. Chromosomes in breast lymphoma. Blood. 2020;136:2848–2849. 33331930 10.1182/blood.2020008964

[154] Drieux F Ruminy P Abdel-Sater A. Defining signatures of peripheral T-cell lymphoma with a targeted 20-marker gene expression profiling assay. Haematologica. 2020;105:1582–1592. 31488561 10.3324/haematol.2019.226647 PMC7271600

[155] Drieux F Ruminy P Sater V. Detection of gene fusion transcripts in peripheral T-cell lymphoma using a multiplexed targeted sequencing assay. J Mol Diagn. 2021;23:929–940. 34147695 10.1016/j.jmoldx.2021.04.013

[156] Carbone A Vaccher E Gloghini A. Hematologic cancers in individuals infected by HIV. Blood. 2022;139:995–1012. 34469512 10.1182/blood.2020005469

[157] Carbone A Gloghini A Serraino D. Immunodeficiency-associated Hodgkin lymphoma. Expert Rev Hematol. 2021;14:547–559. 34044724 10.1080/17474086.2021.1935851

[158] Ambinder RF. Epstein-barr virus-associated post-transplant lymphoproliferative disease. Recent Results Cancer Res. 2021;217:197–207. 33200367 10.1007/978-3-030-57362-1_9

[159] Noy A. Optimizing treatment of HIV-associated lymphoma. Blood. 2019;134:1385–1394. 30992269 10.1182/blood-2018-01-791400 PMC7493463

[160] Ohmoto A Fuji S. Clinical features and treatment strategies for post-transplant and iatrogenic immunodeficiency-associated lymphoproliferative disorders. Blood Rev. 2021;49:100807. 33579543 10.1016/j.blre.2021.100807

[161] Shah N Eyre TA Tucker D. Front-line management of post-transplantation lymphoproliferative disorder in adult solid organ recipient patients—A British Society for Haematology Guideline. Br J Haematol. 2021;193:727–740. 33877688 10.1111/bjh.17421

[162] Alderuccio JP Olszewski AJ Evens AM. HIV-associated Burkitt lymphoma: outcomes from a US-UK collaborative analysis. Blood Adv. 2021;5:2852–2862. 34283175 10.1182/bloodadvances.2021004458 PMC8341354

[163] Eyre TA Caillard S Finel H. Autologous stem cell transplantation for post-transplant lymphoproliferative disorders after solid organ transplantation: a retrospective analysis from the Lymphoma Working Party of the EBMT. Bone Marrow Transplant. 2021;56:2118–2124. 33864020 10.1038/s41409-021-01270-5 PMC8410594

[164] Allred J Bharucha K Özütemiz C. Chimeric antigen receptor T-cell therapy for HIV-associated diffuse large B-cell lymphoma: case report and management recommendations. Bone Marrow Transplant. 2021;56:679–682. 32764581 10.1038/s41409-020-01018-7 PMC7956159

[165] Munshi NC Avet-Loiseau H Anderson KC. A large meta-analysis establishes the role of MRD negativity in long-term survival outcomes in patients with multiple myeloma. Blood Adv. 2020;4:5988–5999. 33284948 10.1182/bloodadvances.2020002827 PMC7724898

[166] Zamagni E Nanni C Dozza L. Standardization of 18F-FDG-PET/CT according to deauville criteria for metabolic complete response definition in newly diagnosed multiple myeloma. J Clin Oncol. 2021;39:116–125. 33151787 10.1200/JCO.20.00386

[167] Zamagni E Nanni C Gay F. MRD evaluation By PET/CT according to deauville criteria combined with multiparameter flow cytometry in newly diagnosed transplant eligible multiple myeloma (MM) patients enrolled in the phase II randomized forte trial. Blood. 2019;134(suppl 1):4321–4321.

[168] Kumar S Paiva B Anderson KC. International Myeloma Working Group consensus criteria for response and minimal residual disease assessment in multiple myeloma. Lancet Oncol. 2016;17:e328–e346. 27511158 10.1016/S1470-2045(16)30206-6

[169] Cohen AD. Myeloma: next generation immunotherapy. Hematology Am Soc Hematol Educ Program. 2019;2019:266–272. 31808859 10.1182/hematology.2019000068 PMC6913481

[170] Munshi NC Anderson LD Jr Shah N. Idecabtagene vicleucel in relapsed and refractory multiple myeloma. N Engl J Med. 2021;384:705–716. 33626253 10.1056/NEJMoa2024850

[171] Berdeja JG Madduri D Usmani SZ. Ciltacabtagene autoleucel, a B-cell maturation antigen-directed chimeric antigen receptor T-cell therapy in patients with relapsed or refractory multiple myeloma (CARTITUDE-1): a phase 1b/2 open-label study. Lancet. 2021;398:314–324. 34175021 10.1016/S0140-6736(21)00933-8

[172] van de Donk N Pawlyn C Yong KL. Multiple myeloma. Lancet. 2021;397:410–427. 33516340 10.1016/S0140-6736(21)00135-5

[173] Gandhi UH Cornell RF Lakshman A. Outcomes of patients with multiple myeloma refractory to CD38-targeted monoclonal antibody therapy. Leukemia. 2019;33:2266–2275. 30858549 10.1038/s41375-019-0435-7 PMC6820050

[174] Chalopin T Vallet N Theisen O. No survival improvement in patients with high-risk multiple myeloma harbouring del(17p) and/or t(4;14) over the two past decades. Br J Haematol. 2021;194:635–638. 33928635 10.1111/bjh.17488

[175] Palumbo A Bringhen S Mateos MV. Geriatric assessment predicts survival and toxicities in elderly myeloma patients: an International Myeloma Working Group report. Blood. 2015;125:2068–2074. 25628469 10.1182/blood-2014-12-615187 PMC4375104

[176] Corre J Perrot A Caillot D. del(17p) without TP53 mutation confers a poor prognosis in intensively treated newly diagnosed patients with multiple myeloma. Blood. 2021;137:1192–1195. 33080624 10.1182/blood.2020008346 PMC7933766

[177] Larocca A Bonello F Gaidano G. Dose/schedule-adjusted Rd-R vs continuous Rd for elderly, intermediate-fit patients with newly diagnosed multiple myeloma. Blood. 2021;137:3027–3036. 33739404 10.1182/blood.2020009507

[178] Stege CAM Nasserinejad K van der Spek E. Ixazomib, daratumumab, and low-dose dexamethasone in frail patients with newly diagnosed multiple myeloma: the hovon 143 study. J Clin Oncol. 2021;39:2758–2767. 33945289 10.1200/JCO.20.03143

[179] Avet-Loiseau H San-Miguel J Casneuf T. Evaluation of sustained minimal residual disease negativity with daratumumab-combination regimens in relapsed and/or refractory multiple myeloma: analysis of POLLUX and CASTOR. J Clin Oncol. 2021;39:1139–1149. 33513030 10.1200/JCO.20.01814 PMC8078259

[180] Bertamini L D’Agostino M Gay F. MRD assessment in multiple myeloma: progress and challenges. Curr Hematol Malig Rep. 2021;16:162–171. 33950462 10.1007/s11899-021-00633-5

[181] Abeykoon JP Murray DL Murray I. Implications of detecting serum monoclonal protein by MASS-fix following stem cell transplantation in multiple myeloma. Br J Haematol. 2021;193:380–385. 33216966 10.1111/bjh.17195

[182] Holthof LC Stikvoort A van der Horst HJ. Bone marrow mesenchymal stromal cell-mediated resistance in multiple myeloma against NK cells can be overcome by introduction of CD38-CAR or TRAIL-variant. HemaSphere. 2021;5:e561. 33898931 10.1097/HS9.0000000000000561 PMC8061681

[183] Depil S Duchateau P Grupp SA. ‘Off-the-shelf’ allogeneic CAR T cells: development and challenges. Nat Rev Drug Discov. 2020;19:185–199. 31900462 10.1038/s41573-019-0051-2

[184] Corre J Munshi NC Avet-Loiseau H. Risk factors in multiple myeloma: is it time for a revision? Blood. 2021;137:16–19. 33024991 10.1182/blood.2019004309 PMC7808011

[185] Cavo M Gay F Beksac M. Autologous haematopoietic stem-cell transplantation versus bortezomib-melphalan-prednisone, with or without bortezomib-lenalidomide-dexamethasone consolidation therapy, and lenalidomide maintenance for newly diagnosed multiple myeloma (EMN02/HO95): a multicentre, randomised, open-label, phase 3 study. Lancet Haematol. 2020;7:e456–e468. 32359506 10.1016/S2352-3026(20)30099-5

[186] Brown S Sherratt D Hinsley S; Myeloma UK Early Phase Clinical Trial Network. MUKnine OPTIMUM protocol: a screening study to identify high-risk patients with multiple myeloma suitable for novel treatment approaches combined with a phase II study evaluating optimised combination of biological therapy in newly diagnosed high-risk multiple myeloma and plasma cell leukaemia. BMJ Open. 2021;11:e046225. 10.1136/bmjopen-2020-046225 PMC7993167 33762245

[187] D’Agostino M Larocca A Offidani M. Octogenarian newly diagnosed multiple myeloma patients without geriatric impairments: the role of age >80 in the IMWG frailty score. Blood Cancer J. 2021;11:73. 33846296 10.1038/s41408-021-00464-w PMC8041817

[188] Stege CAM Nasserinejad K Klein SK. Improving the identification of frail elderly newly diagnosed multiple myeloma patients. Leukemia. 2021;35:2715–2719. 33589752 10.1038/s41375-021-01162-z

[189] Prasad V De Jesús K Mailankody S. The high price of anticancer drugs: origins, implications, barriers, solutions. Nat Rev Clin Oncol. 2017;14:381–390. 28290490 10.1038/nrclinonc.2017.31

[190] Bhattacharya K Bentley JP Ramachandran S. Phase-specific and lifetime costs of multiple myeloma among older adults in the US. JAMA Netw Open. 2021;4:e2116357. 34241627 10.1001/jamanetworkopen.2021.16357 PMC8271356

[191] Blommestein HM Franken MG van Beurden-Tan CHY. Cost-effectiveness of novel treatment sequences for transplant-ineligible patients with multiple myeloma. JAMA Netw Open. 2021;4:e213497. 33779744 10.1001/jamanetworkopen.2021.3497 PMC8008287

